# Impaired tissue homing by the Ikzf3^N159S^ variant is mediated by interfering with Ikaros function

**DOI:** 10.3389/fimmu.2023.1239779

**Published:** 2023-08-17

**Authors:** Jingjie Chang, Motoi Yamashita, Aditya K. Padhi, Kam Y. J. Zhang, Ichiro Taniuchi

**Affiliations:** ^1^ Laboratory for Transcriptional Regulation, RIKEN Center for Integrative Medical Sciences, Yokohama, Kanagawa, Japan; ^2^ Department of Pediatrics and Developmental Biology, Graduate School of Medical and Dental Sciences, Tokyo Medical and Dental University (TMDU), Tokyo, Japan; ^3^ Laboratory for Structural Bioinformatics, RIKEN Center for Biosystems Dynamics Research, Yokohama, Kanagawa, Japan

**Keywords:** IKZF family proteins, lymphocyte homing, CD62L, immune deficiency, heterodimeric interference

## Abstract

AIOLOS, encoded by *IKZF3*, is a member of the IKZF family of proteins that plays an important role in regulating late B-cell differentiation. Human individuals heterozygous for the AIOLOS p.N160S variant displayed impaired humoral immune responses as well as impaired B and T cell development. We have previously reported that a mouse strain harboring an *Ikzf3^N159S^* allele that corresponds to human *IKZF3^N160S^* recapitulated immune-deficient phenotypes, such as impaired B cell development and loss of CD23 expression. In this study, we investigated the effect of the *Ikzf3^N159S^* variant and found that B1a cell development was impaired in *Ikzf3^N159S/N159S^* mice. In addition, CD62L expression was severely decreased in both B and T lymphocytes by the *Ikzf3^N159S^* mutation, in a dose-dependent manner. Mixed bone marrow chimera experiments have revealed that most immunodeficient phenotypes, including low CD62L expression, occur in intrinsic cells. Interestingly, while *Ikzf3^N159S/N159S^* lymphocytes were still present in the spleen, they were completely outcompeted by control cells in the lymph nodes, suggesting that the capacity for homing or retention in the lymph nodes was lost due to the *Ikzf3^N159S^* mutation. The homing assay confirmed severely decreased homing abilities to lymph nodes of *Ikzf3^N159S/N159S^* B and T lymphocytes but selective enrichment of CD62L expressing *Ikzf3^N159S/N159S^* lymphocytes in lymph nodes. This finding suggests that impaired CD62L expression is the major reason for the impaired homing capacity caused by the *Ikzf3^N159S^* mutation. Interestingly, an excess amount of Ikaros, but not Aiolos, restored CD62L expression in *Ikzf3^N159S/N159S^* B cells. Together with the loss of CD62L expression due to Ikaros deficiency, the Aiolos^N159S^ mutant protein likely interferes with Ikaros function through heterodimerization, at least in activating the *Sell* gene encoding CD62L expression. Thus, our results revealed that Aiolos^N159S^ causes some immunodeficient phenotypes *via* the pathogenesis referred to as the heterodimeric interference as observed for Aiolos^G158R^ variant.

## Introduction

Inborn errors of immunity (IEI), also known as primary immunodeficiency disorders (PIDs), are characterized by defects in development or dysfunction of the immune system, resulting in increased susceptibility to infections or dysregulated immune responses (1). IEIs are a heterogeneous group of disorders characterized by a defect in the function of at least one, and often more, components of the immune system (2). Germline variants in single genes often caused IEI, and more than 450 genes have been identified to be related to IEIs (1). Some IEIs are caused by monoallelic variants through haploinsufficiency, negative dominance, or gain of function (GOF). Among these monoallelic variants, missense variants in the genes encoding transcriptional regulators have received attention. For instance, variants of STAT1, c-MYB, and BCL11B, have been isolated from human patients with IEI (3–6).

The IKAROS (IKZF) family members include IKZF1 (Ikaros), IKZF2 (Helios), IKZF3 (Aiolos), IKZF4 (Eos), and IKZF5 (Pegasus), which are critical regulators of hematopoiesis (7, 8). The IKZF family proteins function as either homo- or heterodimer and regulate gene expression in both positive and negative manners (9). Structurally, IKZF family proteins are characterized by four zinc fingers (ZFs) in the middle of the molecule for binding to DNA and two ZFs at the C-terminus for homo- and heterodimerization with IKZF members (10). IKAROS/IKZF1, the first and most studied member of the IKZF family, plays a key role in generating early hematopoietic progenitors and is necessary for lymphoid differentiation (10, 11). In a murine *Ikzf1* mutant allele that removes ZF1, ZF2, and ZF3 that are responsible for DNA binding, a truncated Ikzf1 protein lacking DNA-binding capacity functions as a dominant negative (DN) form. In mice homozygous for this Ikzf1 mutant allele (*Ikzf1^DN/DN^* mice), the generation of lymphoid progenitors is severely damaged, thereby generating almost no mature B and T lymphocytes (7, 8). Natural killer (NK) cell development is also inhibited in *Ikzf1^DN/DN^* mice (12). Thus, studies in mice have revealed the critical roles of Ikzf1 in the hematopoiesis and lymphoid cell development (7, 8, 12–14). Moreover, heterozygous variants of IKZF1 have been reported from human patients with combined immunodeficiency (15–19). IKZF1 creates macromolecular complexes that produce different functional activities in the hematopoietic, lymphopoietic, and non-lymphoid systems (20–24). Among IKZF family proteins, AIOLOS/IKZF3 shows the highest homology to IKAROS and has been confirmed to make heterodimerize with IKAROS (20). However, contrary to *IKZF1*, *IKZF3* is not expressed in hematopoietic stem or progenitor cells. Its expression in the bone marrow is first detected at low levels in pro-B cells and is upregulated as this progress to pre-B cells and mature peripheral B cells (16, 25). Mouse studies have shown that Aiolos promotes activation and maturation of B lymphocytes (25). Recently, a missense mutation in *IKZF3*, *IKZF3^G159R^*, has been identified in a human family with impaired B-cell development (16). Yamashita et al. proposed that B cell deficiency in *IKZF3^+/G159R^* patients is caused by hijacking IKZF1 function by the *IKZF3^G159R^* variant, a pathogenesis referred to as “heterodimeric interference” (16, 26). Another IKZF3 variant encoding AIOLOS^N160S^ was identified in the patients presenting with hypogammaglobulinemia, susceptibility to *Pneumocystis jirovecii* pneumonia, and chronic lymphocytic leukemia (27). In mice harboring *Ikzf3^N159S^* corresponding to the human *IKZF3^N160S^*, certain immune phenotypes observed in human patients, such as loss of follicular T cells and decreased CD23 expression, were shown to be recapitulated (27).

In this study, we further characterized *Ikzf3^N159S^* mutant mice and found CD62L expression was severely reduced in both B and T lymphocytes by Aiolos^N159S^ in a dose-dependent manner. The homing assay confirmed the impaired homing ability of *Ikzf3^N159S^* ^/N159S^ T and B lymphocytes. Selective homing of CD62L expressing *Ikzf3^N159S^* ^/N159S^ cells into the lymph nodes (LNs) suggested that low CD62L expression is a major reason for reduced homing activity. Retroviral transduction of Ikaros, but not Aiolos, into *Ikzf3^N159S^* ^/N159S^ B cells restored CD62L expression, indicating that Aiolos^N159S^ reduces CD62L expression by disturbing the function of Ikaros.

## Materials and methods

### Mice

The *Ikzf3^N159S^* mutant mouse strain was generated by CRISPRCas9–mediated genome editing and has been reported (27). CD45.1 congenic mice and *Rag1-*deficient mice were obtained Jackson Laboratory. All mice were maintained in the animal facility at RIKEN Center for Integrative Medical Sciences, and all animal procedures were in accordance with institutional guidelines for animal care and the protocol approved by the Institutional Animal Care and Use Committee of RIKEN Yokohama Branch (2020–026).

### Antibodies

Anti-Ikaros (MABE912) and anti-Aiolos (MABE911) purchased from Merck were used for western blot experiments. Antibodies and clone names used for flow cytometry were CD43 (S7), BP-1 (BP-1), IgM (R6-60.2), B220 (RA3-6B2), CD11b (M1/70), CD19 (1D3), CD24 (M1/69), CD93 (AA4.1), CD62L (MEL-14), CD44 (IM7), CD4 (RM4-5), CD8a (53-6.7), TCRβ (H57-597), CD3e (17A2), IgD (11-26c.2a), CD23 (B3B4), CD21 (7G6), CD5 (53-7.3), CD45.1 (A20), CD45.2 (104), Ly6G (1A8), Ly6C (HK1.4), CD11c (HL3), CD127 (A7R34) and NK1.1 (PK136) and were purchased from either BD Bioscience or BioLegend.

### Flow cytometry and definition of cell types

Flow cytometry experiments and fluorescence-activated cell sorting were performed on FACSCanto™ II (BD Biosciences) and FACSAria (BD Biosciences), respectively, and were analyzed by FlowJo software (Tree Star). Cell types were defined as follows: B cells (B220^+^CD19^+^), B-1 cells (CD19^hi^B220^lo^), B-2 cells (CD19^lo^B220^hi^), B-1a cells (CD19^hi^B220^lo^CD5^+^), B-1b cells (CD19^hi^B220^lo^CD5^−^), T cells (TCRβ^+^CD3^+^), naïve T cells (CD62L^+^CD44^−^), central memory cells (CD62L^+^CD44^+^), effector memory cells (CD62L^−^CD44^+^), FO B cells (CD19^+^CD21^int^CD23^hi^), MZ B cells (CD19^+^CD21^hi^CD23^lo/-^). B cells in bone marrow, Fr.A (B220^+^CD43^+^Bp-1^−^CD24^−^), Fr.B (B220^+^CD43^+^Bp-1^−^CD24^+^), Fr.C (B220^+^CD43^+^Bp-1^+^CD24^+^), Fr.D (B220^+^CD43^−^AA4.1^+^IgM^−^), Fr.E&F (B220^+^CD43^−^AA4.1^+^IgM^+^) and recirculating B cells (B220^+^CD43^−^AA4.1^−^IgM^+^),dendritic cells (B220^−^TCRβ^−^CD11b^+^CD11c^+^), natural killer cell (B220^−^TCRβ^−^CD11b^+^CD11c^−^NK1.1^+^Ly6G^−^), neutrophils (B220^−^TCRβ^−^CD11b^+^CD11c^−^NK1.1^−^Ly6G^+^), eosinophils (B220^−^TCRβ^−^CD11b^+^CD11c^−^NK1.1^−^Ly6G^−^Ly6C^lo^), monocytes/macrophages (B220^−^TCRβ^−^CD11b^+^CD11c^−^NK1.1^−^Ly6G^−^Ly6C^−^), inflammatory monocytes (B220^−^TCRβ^−^CD11b^+^CD11c^−^NK1.1^−^Ly6G^−^Ly6C^hi^).

### RNA-Seq

RNA-seq was performed using RNA extracted from purified splenic T cells (TCRβ^+^CD4^+^CD44^−^CD127^+^) or naïve B cells (B220^+^CD19^+^) from *Ikzf3^+/+^* (n=2 or 4), *Ikzf3^+/N159S^* (n=2) and *Ikzf3^N159S/N159S^* (n=2) mice. Total RNA was extracted by using Trizol reagent (Invitrogen). The mRNA was obtained by poly(A) selection with NEBNext Poly(A) mRNA Magnetic Isolation Module (E7490). RNA-seq libraries were prepared by NEBNext UltraIIRNA Library Prep Kit for Illumina (E7770) following the manufacturer’s protocol. Briefly, the first and second strands of cDNA were synthesized from mRNA, and were end-repaired, dA tailed and ligated with an adapter (E7335). The adapter-ligated DNA fragments were size selected by dual bead-based size selection using AMPure XP Beads (A63881). The size-selected DNA was amplified by PCR for 11–12 cycles. The purified DNA was diluted to 1 ng and was sequenced with a HiSeqX sequencer (Illumina) at Macrogen.

Trimmomatic is used to trim reads and removing adapter sequences. For analysis of differentially expressed genes (DEGs), RNA-seq reads were mapped to the murine genome (mm10) using HISAT2 (version 2.2.1) with default parameters, and read counts were calculated with HTseq (version2.0.2) using refGene as genome index files (28). Generated read counts were analyzed with the R package edgeR (version 3.38.4) (29). For gene ontology (GO) analysis, downregulated DEGs with false discovery rate (FDR) <0.05 in *Ikzf3^N159S/N159S^* T cells or B cells compared to *Ikzf3^+/+^* controls were extracted and subjected to the analysis. GO enrichment analysis was performed with the R package clusterProfiler (version 4.4.4) (30).

And the ENCODE database showed the Ikaros protein binds to *SELL* gene in human B-Lymphocyte cell line (GM12878 cell line). And Harmonizome databases extract and organize the genes or proteins from publicly available resources (31). In Harmonizome databases, we search and confirm which down-regulated genes of RNA-seq data are the target genes of the IKZF1 transcription factor in ChIP-seq datasets, and then verified them in the ENCODE database.

### Plasmid preparation

We constructed retroviral vectors for generating retroviruses to infect mouse B cells. The entire coding region of mouse Ikaros/*Ikzf1* (NM_001025597.3) was amplified from cDNA prepared from wild-type splenic cells by RT-PCR and was cloned into pCR2.1^®^-TOPO vector by TOPO TA Cloning Kit (Invitrogen). After confirming the sequence, the cDNA fragment was cloned into the pMigR-DsRED plasmid, which was generated by ourselves *via* replacing the GFP sequence with DsRED in the pMigR1 plasmid (Addgene Plasmid #27490). Similarly, cDNA encoding mouse Aiolos/*Ikzf3* (NM_011771.1) was amplified by RT-PCR and was cloned into pMigR1 plasmid. Primers sequences were following: *Ikzf1*-Forward : CCCAGATCTAGACAATGGATGTCG-ATGAGG, *Ikzf1*-Reverse : CCCCTCGAGGGTTTAGCTCAGGTGGTAAC, *Ikzf3*-Forward : CCCGCGGCCGCCGGCGACATGGAAGATATAC, *Ikzf3*-Reverse : CCCCTC GAGTGCTCACTTCAACATGGCTC

### Retroviral transduction into B cells

Plate-E packaging cell line was cultured in DMEM medium (Gibco) supplemented with 10% FBS (Hyclone), 1% penicillin/streptomycin (Gibco), 10 µg/ml Blasticidin (Gibco) and 1 µg/ml Puromycin (Gibco) at 37°C with 5% CO_2_. Plate-E cells (2*10^5^/ml in a 6-well dish) were transfected with 2 µg of the retroviral vector using a FuGENER HD Transfection Reagent (Promega) at a 3:1 ratio (FuGENER HD: DNA mass per well). After 48h, the supernatant was collected. B cells from WT and *Ikzf3^N159S/N159S^* mice were isolated using B cell Microbead (Miltenyi Biotec) with purity over 92%. 10^6^ of these B cells were stimulated with 10 µg/ml LPS (Sigma) in RPMI medium (Gibco), supplemented with 10% FBS, 50 µmol 2-mercaptoethanol (Sigma), 1mM sodium pyruvate (Gibco), 1% penicillin/streptomycin (Gibco) and 1% MEM (Gibco). Two days after stimulation, viral supernatant was added with 20 mg/ml polybrene (Greiner bio-one) to the cells in a 24-well plate. To enhance transduction efficiency, cells were subjected to a round of centrifugation with vector-containing medium for 90min at 2000rpm and 32°C, separated by a 2-hour interval in which cells were cultured in medium without viral particles. Two days after infection, the cells were FACS analyzed for various purposes as indicated.

### Generation of bone marrow chimeras

For the generation of bone marrow chimeras, bone marrow (BM) cells were prepared from CD45.2 WT or *Ikzf3^N159S/N159S^* mice and CD45.1 wild type mice. Those donor-derived CD45.2 bone marrow cells were mixed 1:1 with bone marrow cells from CD45.1^+^ wild type cells and were transferred intravenously into sub-lethally (950 rad) irradiated *Rag1^−/−^* recipients. The recipient mice were analyzed 8–12 weeks after bone marrow injection.

### Homing assay

The homing assay was performed as described (32–35) with slight modifications. Single splenic cell suspensions were prepared from CD45.2 WT or *Ikzf3^N159S/N159S^* mice and CD45.1 WT mice. Equal numbers (3-5 × 10^7^) of CD45.2 WT or *Ikzf3^N159S/N159S^* cells and CD45.1 WT cells were mixed and were intravenously co-injected into CD45.1/2 recipient mice. An aliquot of the injected cells mixture was analyzed by flow cytometry to confirm the injected ratio of CD45.1^+^ and CD45.2^+^ (Ri) in B220^+^CD19^+^ or TCRβ^+^CD3^+^ population. After 24h of migration, single-cell suspensions of blood and organs were prepared and were stained with antibodies. The ratio of CD45.1^+^CD45.2^−^ and CD45.2^−^CD45.2^+^ (Ro) was determined by flow cytometry. The ratio of CD45.1^+^CD45.2^−^ and CD45.2^−^CD45.2^+^ cells in B220^+^CD19^+^ or TCRβ^+^CD3^+^ population within individual injection mixture (Ri) and organs (Ro) was measured, and the homing assay results were presented as the ratio of Ro/Ri in each tissue.

### Template selection and homology modeling

Given the absence of experimentally determined three-dimensional structure for human AIOLOS, a homology model of the four N-terminal zinc fingers was constructed. Initially, a template search was performed using BLASTP against the RCSB protein data bank (PDB) with the human AIOLOS sequence as the query, leading to the identification of potential template sequences. After careful evaluation, a template fragment of human PR/SET domain 9 (PRDM9c) with a PDB ID of 5V3G, encompassing six zinc fingers, was selected (36–38). The choice was based on its sequence identity of 38%, absence of gaps (0% gap), 55% positive residues, and a high BLAST bit score of 224.

To generate the homology models, MODELLERv9.17 was employed (39). Initially, the target and template sequences were aligned using MODELLER’s align2d command, which employs global dynamic programming with a linear gap penalty function to align the two profiles. Subsequently, 20 three-dimensional models of human AIOLOS with four N-terminal zinc fingers and DNA were built using MODELLER. Among the generated models, the one with the lowest DOPE (Discrete Optimized Protein Energy) score, as well as visual inspection, was chosen for further analysis (40). This selected model represents the complex of human AIOLOS with four N-terminal zinc fingers, cognate DNA sequence of AIOLOS, and zinc ions, and served as the starting structure for subsequent molecular dynamics simulations and analyses.

### Molecular dynamics simulations and analyses

To investigate the behavior of the N160S as compared to wild-type (WT) and G159R-mutant structures, we first prepared the N160S-mutant by mutating the N160 to S160 *in silico*, while preserving the secondary structure. Subsequently, molecular dynamics (MD) simulations were performed. The initial step involved the addition of hydrogen atoms to all the systems. Subsequently, these structures were solvated using the three-point transferable intermolecular potential (TIP3P) water model in an octahedral box, while maintaining a distance of approximately 10 Å between the protein-DNA complex surface and the box boundary (41). The systems were then neutralized by introducing counter ions, and the simulations were performed using GROMACS 5.1.2 with the ff14SB forcefield (42, 43). The MD simulations commenced with an energy minimization phase, consisting of 50,000 steps, followed by a gradual heating process from 0 to 300 K over 500 ps. Subsequently, a constant temperature equilibration was performed for 3000 ps at 300 K. To ensure velocity stability, the Parrinello-Rahman barostat pressure coupling method was employed. The production runs for the WT, N160S and G159R mutants lasted 500 ns, employing a periodic boundary condition in the NPT ensemble. Berendsen temperature coupling with modifications was utilized to maintain a constant temperature of 300 K and a pressure of 1 atm. The leap-frog integrator and the Verlet cut-off schemes were employed during the simulations (44). To preserve bond lengths, the LINCS algorithm was used, while the Particle-mesh Ewald method was employed for the calculation of electrostatic forces. In these calculations, Fourier grid spacing and Coulomb radius values of 0.16 nm and 1.4 nm, respectively, were utilized. The standard van der Waals interactions were set to 1.4 nm. To analyze the MD simulated trajectories of the three systems, several key metrics were calculated. The root means square deviation (RMSD), root mean square fluctuation (RMSF), and radius of gyration (Rg) were determined, for which the GROMACS utilities, namely gmx rms, gmx rmsf, and gmx gyrate, were employed for these calculations respectively, providing essential insights into the structural stability and flexibility of the systems. The MD simulation trajectories were visualized using Visual Molecular Dynamics (VMD) (45). Molecular visualization and figure generation were accomplished using PyMOL (http://www.pymol.org).

## Results

### Impaired B1a cell development in Aiolos^N159S^ mutant mice

**Figure 1:**
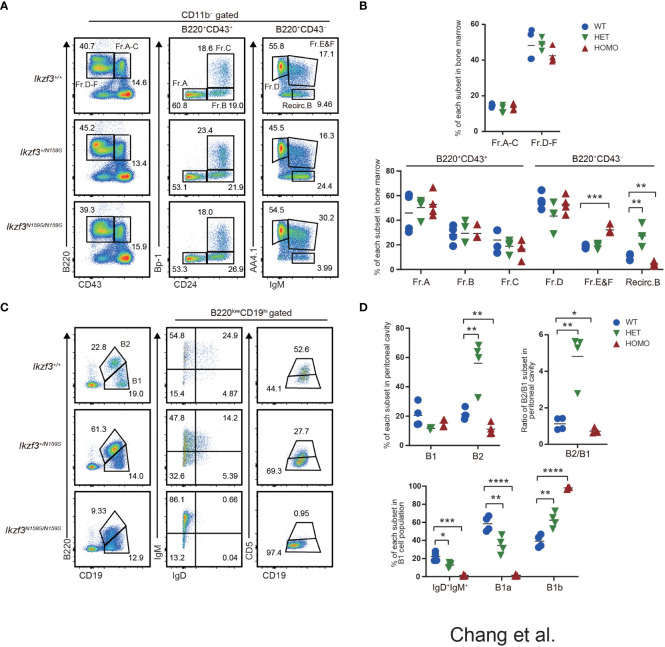
Impaired B1a cell development in *Ikzf3^N159S/N159S^* mice. **(A)** Flow cytometric analysis of bone marrow cells from 6-12 weeks age mice with indicated genotypes. CD11b^−^B220^+^ cells can be resolved into Fr.A-C (B220^+^CD43^+^) and Fr.D-E (B220^+^CD43^−^) subsets. Fr.A-C and Fr.D-F subsets are divided into six subsets: Fr.A, Bp1^−^CD24^−^; Fr.B, Bp-1^−^CD24^+^; Fr.C, Bp-1^+^CD24^+^; Fr.D, AA4.1^+^IgM^−^; Fr.E&F, AA4.1^+^IgM^+^; Recirc.B, AA4.1^−^IgM^+^. Numerical values in the plots indicate percentages of each subpopulation. **(B)** Graphs show the frequencies of B cell subsets in the bone marrow (n=3). The horizontal lines indicate the mean values from at least three different mice per group. **(C)** Flow cytometric analyzes of B1 (CD19^hi^B220^lo^) and B2 (CD19^lo^B220^hi^) cell population in the peritoneal cavity, using the indicated markers. CD19^hi^B220^lo^CD5^+^ (B1a) and CD19^hi^B220^lo^CD5^−^ (B1b) of the peritoneal cavity were stained. FACS analysis of B1 cell population from the peritoneal cavity stained for IgM and IgD. Numerical values in the plots indicate percentages of each subpopulation. **(D)** Frequencies of B1, B2 population (top-left) and the ratio of B2/B1 (top-right) in the peritoneal cavity from *Ikzf3^+/+^*, *Ikzf3^+/N159S^*, *Ikzf3^N159S/N159S^* mice are shown (n=3). Additionally, the graph below shows the frequencies of IgM^+^IgD^+^, B1a and B1b subpopulations from total B1 cells (n=3). Statistically significant differences (Student’s unpaired, two-tailed t test) between the groups are shown. *, P < 0.05; **, P < 0.01; ***, P < 0.001; ****, P < 0.0001.

We have previously reported that the knock-in mouse model carrying the *Ikzf3^N159S^* mutant allele generating the Aiolos^N159S^ missense mutant protein, which corresponds to the human AIOLOS^N160S^ variant, recapitulated the abnormal B and T lymphocyte development similarly to what was observed in the patients with *IKZF3^N160S^* variant. B cell frequencies in peripheral blood (PBL) was decreased in *Aiolos^N159S/N159S^* mice (27). In addition, we showed that the expressions of IgM, IgD, and CD23 significantly decreased in B lymphocyte populations in both *Ikzf3^+/N159S^* and *Ikzf3^N159S/N159S^* mice (27). To clarify the developmental stages of B cell development impaired by the *Aiolos^N159S^* mutation, we examined early B cell development in the bone marrow according to the criteria of Fractions A to G defined by Hardy (46). No significant differences were observed in the frequencies of fractions A-C among *Ikzf3^+/+^*, *Ikzf3^+/N159S^* and *Ikzf3^N159S/N159S^* mice (**Figures 1A, B**). However, in the CD11b^−^B220^+^CD43^−^ population, AA4.1^+^IgM^+^ cells corresponded to Fr. E & F were increased in *Ikzf3^N159S/N159S^* mice with a decrease in recirculating B cells, which were defined as CD11b^−^B220^+^CD43^−^AA4.1^−^IgM^+^ (**Figures 1A, B**). In contrast, recirculating B cells increased in *Ikzf3^+/N159S^* mice (**Figures 1A, B**), which is consistent with the tendency of increased splenic B cell frequency in *Ikzf3^+/N159S^* mice (27). Therefore, an increase in Fr. E & F in *Ikzf3^N159S/N159S^* mice might reflect a relative increase due to the low frequency of recirculating B cells, rather than the accumulation of immature B cells by developmental blockade.

B-lymphocytes are divided into B1 and B2 subtypes. B1 cells are an important part of the innate immune response, and spontaneously secrete polyreactive IgM antibodies (47, 48). In mice, B1 cells differ from conventional B2 B cells in that they do not express CD21 and CD23 and express only low levels of B220 (B220^lo^CD19^hi^) and IgD, but high levels of IgM. B1 cells are abundant in both peritoneal and pleural cavities and can be further divided into B1a (B220^lo^CD19^hi^CD5^+^) and B1b (B220^lo^CD19^hi^CD5^−^) cells (47, 48). CD5^+^ B1a cells are thought to be long-lived, self-renewing cells that predominantly reside in these pleural and peritoneal cavities, where they produce natural polyreactive IgM antibodies with a biased, autoreactive repertoire (49).

Given the reduction in B2 cell numbers in *Ikzf3^N159S/N159S^* mice, we examined whether B1 cell development was impaired by the *Ikzf3^N159S^* mutation. Consistent with the tendency of splenic B2 cell numbers (27), the frequency of B2 cells, defined as CD19^lo^B220^hi^ cells in the peritoneal cavity of *Ikzf3^+/N159S^* and *Ikzf3^N159S/N159S^* mice, increased and decreased, respectively, thereby increasing and decreasing the B2 to B1 ratio (**Figures 1C, D**). B2 cells in the peritoneal cavity of both mutants also showed decreased IgD and CD23 expression (**Figure S1A**), as observed in the spleen (27). In the CD19^hi^B220^lo^ B1 cell population, the expression level of IgD was also decreased by the *Ikzf3^N159S^* mutation in a dose-dependent manner, and IgD^+^ cells were hardly detected in *Ikzf3^N159S/N159S^* mice (**Figures 1C, D**). B1a cells, defined as CD19^hi^B220^lo^CD5^+^ cells, were significantly decreased in the peritoneal cavities of *Ikzf3^+/N159S^* and *Ikzf3^N159S/N159S^* mice (**Figures 1C, D**). Since CD5 is the only marker to distinguish B1a and B1b cells, loss of B1a cell population could be either caused by loss of CD5 expression from B1a cells or developmental block of B1a cells. In order to distinguish whether CD5 expression was lost in B1a cells or whether B1a cell development was inhibited by Aiolos^N159S^, we examined CD5 expression in other cells such as T lymphocytes. In the spleen, the frequency of CD5^+^ T cells were decreased in *Ikzf3^+/N159S^* mice with severer reduction in CD8^+^ T cells (**Figure S1B**). Primary T cell development in the thymus is divided into four stages according to CD4 and CD8 co-receptor expression: most immature CD4^−^CD8^−^ (DN) stage is flowed by CD4^+^CD8^+^ (DP) precursor stage, and DP precursor is maturated into CD4^+^CD8^−^ (CD4 SP) or CD4^−^CD8^+^ (CD8 SP) stage after positive selection. In thymic T cells, the frequency of CD5 expressing cells was significantly decreased in the CD4^+^CD8^+^ (DP) and CD4^−^CD8^+^ (CD8 SP) subsets and increased in the CD4^−^CD8^−^ (DN) subset (**Figure S1C**). This indicated that *Ikzf3^N159S^* mutation disturbed CD5 expression during the DN to DP stages in the thymus, specifically in the CD8 SP population. However, CD5 expression was not completely lost, suggesting that the loss of CD5^+^ B1a population was likely caused by developmental inhibition rather than a loss of CD5 expression.

### Impaired CD62L expression on T and B cells by Aiolos^N159S^ variant

**Figure 2:**
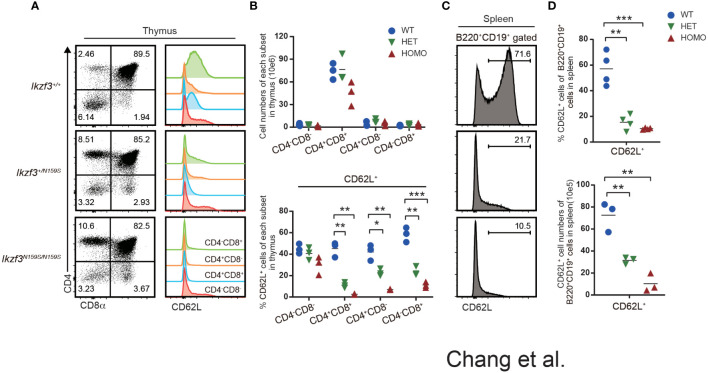
Low CD62L expression on T and B cells by Aiolos^N159S^ variant. **(A)** Flow cytometric analyses of thymocytes from *Ikzf3^+/+^*, *Ikzf3^+/N159S^*, *Ikzf3^N159S/N159S^* mice, at age of 6-12 weeks. CD4 and CD8a were used to stain DN (CD4^−^CD8^−^), DP (CD4^+^CD8^+^), CD4 SP (CD4^+^CD8^−^), CD8 SP (CD4^−^CD8^+^) thymocyte subsets. CD62L expression levels on each thymocyte subsets are adjacently shown as histograms. **(B)** Graphs show cell numbers of CD4^−^CD8^−^, CD4^+^CD8^+^, CD4^+^CD8^−^ and CD4^−^CD8^+^ thymocytes (top) and the percentage of CD62L^+^ cells within each subset (bottom) (n=3). The horizontal lines indicate the mean values of at least three different mice per group. **(C)** Flow cytometric analyzes of CD62L expression on splenic B (B220^+^CD19^+^) cells. **(D)** Graphs show the frequency (top) and cell number (bottom) of CD62L^+^ cells from splenic B cell population from *Ikzf3^+/+^*, *Ikzf3^+/N159S^*, *Ikzf3^N159S/N159S^* mice (n=3). Statistically significant differences (Student’s unpaired, two-tailed t test) between the groups are shown. *, P < 0.05; **, P < 0.01; ***, P < 0.001.

Although just from a single patient, RNA-seq analyses of naïve B cell from such a patient carrying *IKZF3^N160S^* variant showed lower expression of *SELL* gene which encodes CD62L (27). In *Ikzf3^N159S/N159S^* mice, circulating T cells in the peripheral blood, particularly CD8^+^ T cells, showed decreased CD62L expression (27). We then next examined the developmental stages that initiate low CD62L expression during T cell development. In primary T cell development of thymus, flow cytometry analyses detected low CD62L expression of thymocytes of *Ikzf3^+/N159S^* and *Ikzf3^N159S/N159S^* mice from CD4^+^CD8^+^ (DP) stage onward, while CD62L expression on CD4^−^CD8^−^ DN thymocytes was comparable (**Figures 2A, B**). In mice, RNA-seq data available in the ImmGen database showed that the expression of *Ikzf3* gene is initiated from the DN to DP transition, whereas the *Ikzf1* gene is expressed earlier in the DN1 stage (**Figure S2A**). Thus, low CD62L and CD5 expression was correlated with the expression of *Ikzf3^N159S^* gene, suggesting that the Aiolos^N159S^ mutant protein quickly suppresses CD62L expression in a dominant-negative form. Given that CD62L is expressed in mature B cells, we also examined CD62L expression in splenic B220^+^ CD19^+^ B cells and found that expression of CD62L is heavily decreased in *Ikzf3^+/N159S^* and *Ikzf3^N159S/N159S^* mice (**Figures 2C, D**). Furthermore, B220^+^CD19^+^ cells in the peripheral blood and peripheral LN (pLN), and mesenteric LN (mLN), and B2 and B1 cells in the peritoneal cavity showed decreased CD62L expression in *Ikzf3^N159S/N159S^* mice (**Figures S2B, C**). Interestingly, the frequency of CD62L^+^ B cells varied in secondary lymphoid tissues of *Ikzf3^+/N159S^* mice.

### Cell intrinsic phenotypes of Ikzf3^N159S/N159S^ cells assessed by mixed bone marrow chimera

**Figure 3:**
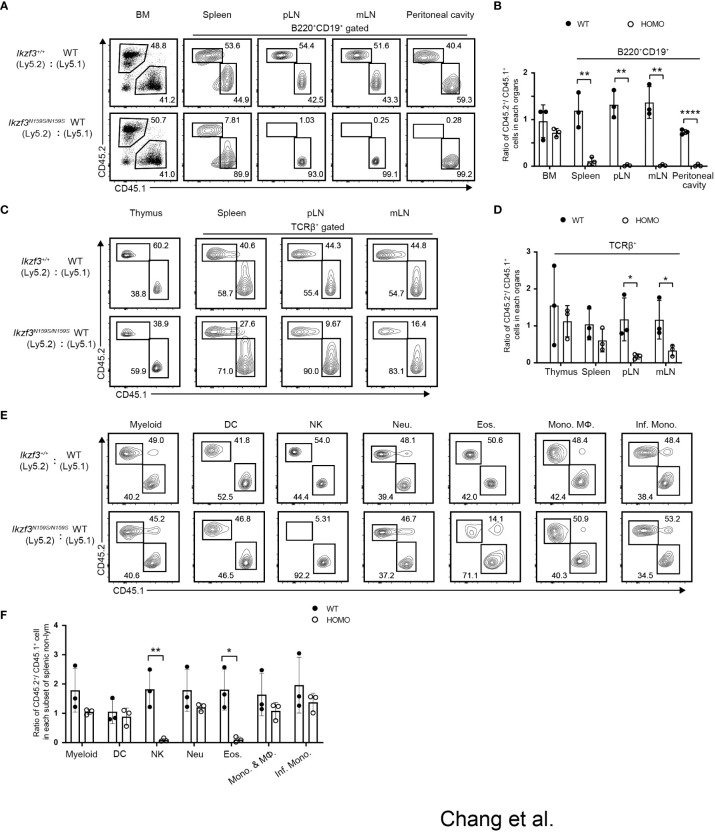
Cell intrinsic phenotypes by the Aiolos^N159S^ variant assessed by mixed bone marrow chimera experiment. **(A)** Flow cytometric analyses show the percentages of CD45.1^+^ (WT) versus CD45.2^+^ (*Ikzf3^+/+^* or *Ikzf3^N159S/N159S^*) donor cells in total bone marrow and in B cell population (B220^+^CD19^+^) from spleen, pLN, mLN and peritoneal cavity, after >8 weeks post-transplantation. **(B)** Ratios of CD45.2^+^/CD45.1^+^ cells in total bone marrow and B cell populations (B220^+^CD19^+^) from spleen, pLN and mLN, after >8 weeks post-transplantation (n=3). Each dot represents data from one recipient mouse. Data are presented as the mean ± SD, n = 3 per group. **(C)** Flow cytometric analyzes show the percentages of CD45.2 and CD45.1 donor cells in total cells of thymus and T cells (TCRβ^+^) from spleen, pLN and mLN, after >8 weeks post-transplantation. **(D)** Ratios of CD45.2^+^/CD45.1^+^ cells in total thymus and in T cells (TCRβ^+^) from spleen, pLN, mLN and peritoneal cavity, after >8 weeks post-transplantation. **(E)** Flow cytometric analyzes show percentage of CD45.2^+^ and CD45.1^+^ donor cells in splenic CD11b^+^CD11c^−^ cell, dendritic cell (DC), natural killer cell (NK), neutrophil (Neu.), eosinophil (Eos.), Monocyte (Mono.), Macrophage (MΦ) and Inflammatory monocyte (Inf. Mono.) subsets, after >8 weeks post-transplantation. **(F)** Ratios of CD45.2^+^/CD45.1^+^ cells in each splenic myeloid cell subset (CD11b^+^CD11c^−^, DC, NK, Neu., Eos., Mono. & MΦ and Inf. Mono.) after >8 weeks post-transplantation (n=3). Statistically significant differences (Student’s unpaired, two-tailed t test) between the groups are shown. *, P < 0.05; **, P < 0.01; ****, P < 0.0001.

We observed that the differentiation of T and B lymphocytes, NK1.1^+^ natural killer (NK) cells, and eosinophils (Eos) was impaired with the loss of certain markers such as CD23 and CD62L in *Ikzf3^N159S/N159S^* mice (27). To address whether these B, T, and myeloid cell phenotypes occur in a cell-intrinsic manner and to test the developmental potency of progenitors under competitive settings, we performed mixed bone marrow (BM) chimera experiments. CD45.1^+^ bone marrow cells were mixed with CD45.2^+^ bone marrow cells prepared from either *Ikzf3^+/+^* or *Ikzf3^N159S/N159S^* mice in equal proportions and transplanted into sublethally irradiated Rag1 KO recipient mice (**Figure S3A**). Reconstitution of hematopoiesis in recipients was analyzed 8 weeks after transplantation using flow cytometry. In the total bone marrow cells, the CD45.2^+^/CD45.1^+^ ratio was close to 1:1 in recipients injected with CD45.1^+^ *Ikzf3^+/+^* and CD45.2^+^ *Ikzf3^N159S/N159S^* BM cells (**Figures 3A, B**). However, during early B cell development, the percentage of CD45.2^+^ *Ikzf3^N159S/N159S^* cells increased in Fr. E and F (**Figures S3B, C**), suggesting that mature B cells in the bone marrow were retained or accumulated in the presence of Aiolos^N159S^. In B220^+^CD19^+^ B cell population in peripheral lymphoid tissues, the percentage of CD45.2^+^ *Ikzf3^N159S/N159S^* cells was significantly decreased to approximately 10% in spleen and was further reduced to less than 1% in pLN, mLN, and peritoneal cavity, comparing with CD45.2^+^ *Ikzf3^+/+^* cells (**Figures 3A, B** and **S3D**).

In the TCRβ^+^ T cell population, CD45.2^+^ *Ikzf3^N159S/N159S^* cells were significantly decreased in the peripheral and mesenteric lymph nodes compared to CD45.2^+^ *Ikzf3^+/+^* cells (**Figures 3C, D** and **S3E**). In NK and myeloid lineage cells, the frequency of CD45.2^+^ *Ikzf3^N159S/N159S^* cells was lower, specifically in CD11b^+^NK1.1^+^ NK cells and CD11b^+^Ly6G^−^Ly6C^lo^ Eos cells. (**Figures 3E, F** and **S3F, G**). These observations indicate that developmental defects of NK and Eos by the *Ikzf3^N159S^* mutation were caused in a cell-intrinsic manner and developmental potency to other myeloid-lineage cells, at least cell types we examined, were not affected by the *Ikzf3^N159S^* mutation.

**Figure 4:**
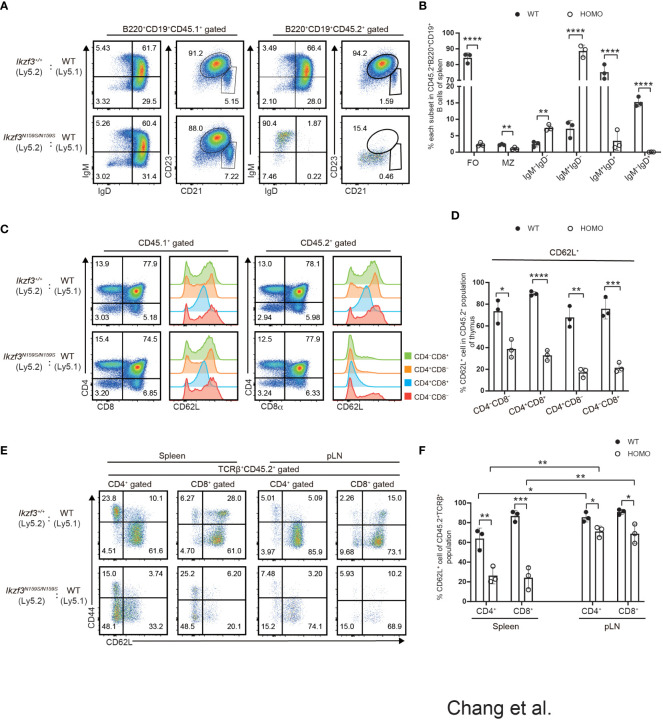
Cell intrinsic defect of CD62L and CD23 expression by the Aiolos^N159S^ variant. **(A)** Flow cytometric analyses show IgM, IgD, CD21 and CD23 expression level on B220^+^CD19^+^ splenic B cells from CD45.1^+^ (WT) versus CD45.2^+^ (*Ikzf3^+/+^* or *Ikzf3^N159S/N159S^*) donor cells, after >8 weeks post-transplantation. **(B)** Frequencies of splenic follicular B cell (CD21^int^CD23^hi^, FO), marginal zone B cell (CD21^hi^CD23^lo/−^, MZ), IgM^−^IgD^−^, IgM^+^IgD^−^, IgM^+^IgD^+^ and IgM^−^IgD^+^ subsets from CD45.2^+^ donor cells are shown (n=3). Each dot represents data from one recipient. Data are presented as the mean ± SD, n = 3 per group. **(C)** Flow cytometric analyses of CD4 and CD8a subsets of CD45.1^+^ or CD45.2^+^ donor cells from WT and Homo group, after >8 weeks post-transplantation. CD62L expression levels on each subset (CD4^−^CD8^−^, CD4^+^CD8^+^, CD4^+^CD8^−^, CD4^−^CD8^+^) are adjacently shown as histograms. **(D)** Graph shows the frequencies of CD62L^+^ of each thymocyte subset from CD45.2^+^ donor cells (n=3). **(E)** Flow cytometric analyses of CD44 and CD62L expression of CD45.2^+^ T cells in spleen and pLN (TCRβ^+^CD4^+^, TCRβ^+^CD8^+^), after >8 weeks post-transplantation. **(F)** Frequencies of CD62L^+^ cells from CD45.2^+^ T cell subsets in spleen and pLN are shown. Statistically significant differences (Student’s unpaired, two-tailed t test) between the groups are shown. *, P < 0.05; **, P < 0.01; ***, P < 0.001; ****, P < 0.0001.

Next, we examined whether the impaired expression of surface proteins caused by the *Ikzf3^N159S^* mutation occurs in a cell-intrinsic or cell-extrinsic manner. In recipients transplanted with CD45.1^+^ *Ikzf3^+/+^* and CD45.2^+^ *Ikzf3^N159S/N159S^* BM cells, the expressions of IgD, CD23, and CD21 in the splenic B cell population were heavily reduced, specifically in CD45.2^+^ *Ikzf3^N159S/N159S^* cells (**Figures 4A, B**). Similarly, CD62L expression was significantly decreased, specifically in CD45.2^+^ *Ikzf3^N159S/N159S^* thymocytes (**Figures 4C, D**). These results indicated that the Aiolos^N159S^ mutant protein inhibits the expression of IgD, CD23, CD21, and CD62L in a cell-intrinsic manner. Interestingly, the frequency of CD62L^+^ cells in each thymocyte subset was higher in these recipients than in the non-irradiated control mice (**Figures 2A, B** and **4C, D**). This suggests that the reconstitution of T cell development in the thymic environment in irradiated recipients may induce CD62L. It is noteworthy that the frequency of CD62L^+^ in CD4^−^CD8^−^ DN subset was already lower in CD45.2^+^ *Ikzf3^N159S/N159S^* thymocytes compared with CD45.2^+^ *Ikzf3^+/+^*, suggesting that a small amount of Aiolos^N159S^ may shortly inhibit CD62L expression after induction in DN cells (**Figures 4C, D**). As expected, CD62L expression was severely inhibited in T cells from the DP thymocytes stage onward and in the spleen (**Figures 4E, F**). However, in pLNs, although the CD45.2^+^ *Ikzf3^N159S/N159S^* T cells comprised a small population, the frequency of CD62L^+^ cells was comparable to that of control cells, similar to CD45.2^+^ *Ikzf3^N159S/N159S^* CD4 T cells in mLN (**Figures 4E, F** and **S4E, F**).

### Reduced homing ability of Aiolos^N159S/N159S^ T and B cells

**Figure 5:**
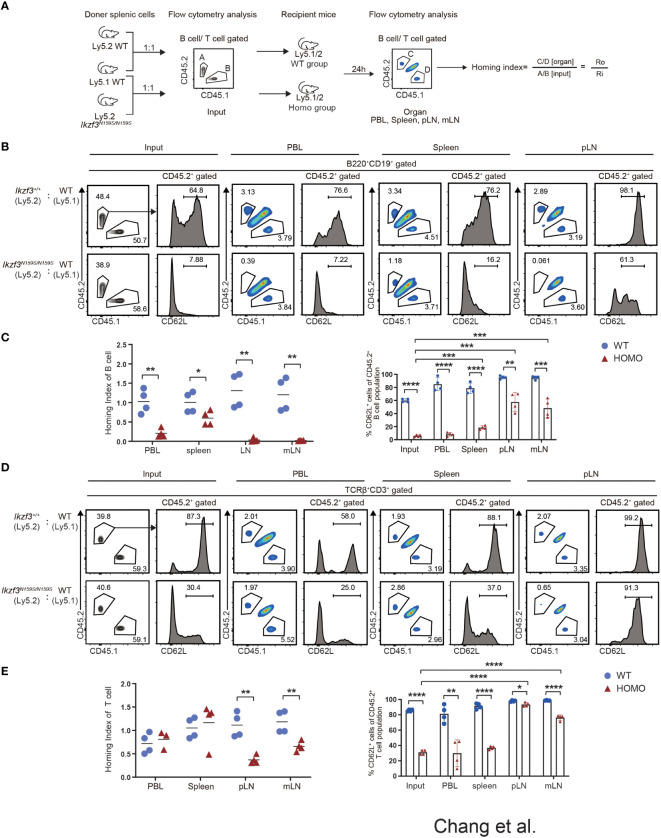
Impaired Homing ability of *Ikzf3^N159S/N159S^* lymphocytes. **(A)** Ly5.2 (CD45.2^+^) *Ikzf3^+/+^* (WT group) or *Ikzf3^N159S/N159S^* (Homo group) splenic cells were mixed with Ly5.1 (CD45.1^+^) wild type (WT) splenic cells and injected into sub-lethally irradiated CD45.1^+^CD45.2^+^ recipient mice. Flow cytometry was used to assess the input ratio (Ri) of CD45.2^+^CD45.1^−^ and CD45.2^−^CD45.1^+^ in B220^+^CD19^+^ or TCRβ^+^CD3^+^ population before injection. After 24h post-injection, flow cytometric analyzes shows ratios of CD45.2^+^CD45.1^−^ and CD45.2^−^CD45.1^+^ donor cells in B or T cell populations from various organs (Ro). The ratio of Ro and Ri is analyzed as the homing index. **(B)** Flow cytometric analyzes shows ratios of CD45.2^+^ and CD45.1^+^ cells in B220^+^CD19^+^ B cells, from input and 24h post-transplanted organs (PBL, spleen, and pLN), alongside CD62L expression levels on CD45.2^+^ B cells. **(C)** B cell homing indexes of PBL, spleen, pLN, and mLN, from WT and Homo groups, are shown on the left. The horizontal lines indicate the mean values of at least three mice per group. The frequencies of CD62L^+^ cells in CD45.2^+^ B cell population from input and 24h post-transplanted organs (PBL, spleen, pLN, and mLN) are shown on the right (n=4). Each dot represents data from one recipient mouse. Data are presented as the mean ± SD, n = 4 per group (right). **(D)** Flow cytometry analyses show ratios of CD45.2^+^ and CD45.1^+^ cells in TCRβ^+^CD3^+^ T cells, from input and 24h post-transplanted organs (PBL, spleen, and pLN), alongside CD62L expression levels on CD45.2^+^ T cells. **(E)** T cell homing indexes of PBL, spleen, pLN, and mLN, from WT and Homo groups, are shown on the left. The frequencies of CD62L^+^ cells from CD45.2^+^ T cell population from input and 24h post-transplanted organs (PBL, spleen, pLN, and mLN) are shown on the right (n=4). *, P < 0.05; **, P < 0.01; ***, P < 0.001; ****, P < 0.0001.

Our mixed bone marrow chimera experiments clearly showed a greater reduction in the frequency of CD45.2^+^ *Ikzf3^N159S/N159S^* lymphocytes in the LNs than in the spleen, whereas the frequency of CD62L^+^ T cells increased in the LNs. Given the important role of CD62L in lymphocyte homing to lymphoid tissue, we compared the homing abilities of *Ikzf3^+/+^* and *Ikzf3^N159S/N159S^* lymphocytes. Total splenocytes from CD45.2^+^ *Ikzf3^+/+^* or CD45.2^+^ *Ikzf3^N159S/N159S^* mice and CD45.1^+^ control mice were mixed in a 1 to 1 ratio and intravenously injected into CD45.1^+^CD45.2^+^ recipient mice (**Figure 5A**). After 24 hours, the frequencies of CD45.1^+^ and CD45.2^+^ cells in the PBL, spleen, and LNs were analyzed by flow cytometry. The ratio of CD45.1^+^CD45.2^−^ and CD45.2^−^CD45.2^+^ in B220^+^CD19^+^ or TCRβ^+^CD3^+^ population in recipient organs (Ro) and that in injection mixture (Ri) were used to calculate the homing index as the ratio of Ro/Ri (**Figure 5A**). In recipients injected with CD45.1^+^ control cells and CD45.2^+^ *Ikzf3^+/+^*, the homing index was approximately one in all tissues examined (**Figures 5B–E** and **S5A**). In contrast, in recipients injected with CD45.1^+^ control cells and CD45.2^+^ *Ikzf3^N159S/N159S^* cells, the homing index in the B cell compartment was lower than 0.2 in the PBL, although it remained around 0.5 in spleen, and CD45.2^+^ *Ikzf3^N159S/N159S^* B cells were hardly detected in the LNs (**Figures 5B, C** and **S5A**). In the T cell population of the above recipients, the homing index was decreased in the pLN and mLN, but not in the PBL and spleen (**Figures 5D, E** and **S5A**). These observations indicate that the homing ability to the LNs was impaired in *Ikzf3^N159S/N159S^* B and T lymphocytes. As expected from analyses of mixed bone marrow chimeras, the frequency of CD62L^+^ cells in *Ikzf3^N159S/N159S^* B and T cell populations was significantly increased in pLN and mLN from the input, although these were still lower than those in CD45.2^+^ *Ikzf3^+/+^* cells (**Figures 5B–E** and **S5A**). These results revealed that *Ikzf3^N159S/N159S^* B and T lymphocytes were outcompeted by control cells in homing to the LNs. Selective accumulation of CD62L^+^ *Ikzf3^N159S/N159S^* lymphocytes in LNs revealed that *Ikzf3^N159S/N159S^* lymphocytes expressing CD62L retained their homing activity to some extent, suggesting that the Aiolos^N159S^ mutation impaired the homing ability of lymphocytes, mainly *via* disturbing the CD62L expression.

### Low expression of putative IKZF1 target genes by Aiolos^N159S^ variant

**Figure 6:**
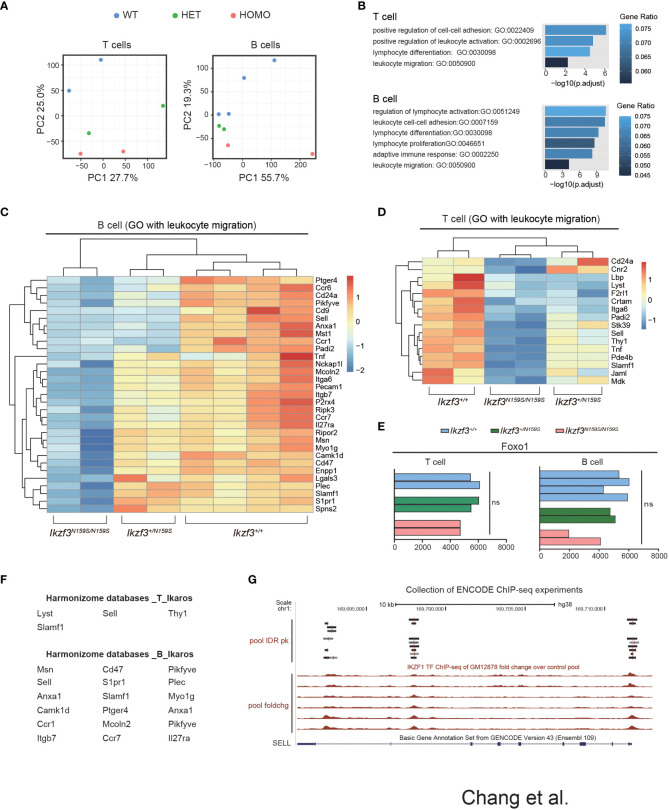
Dysregulated genes by Aiolos^N159S^ revealed by transcriptome analyses. **(A)** Principal component analysis (PCA) of the T and B cells transcriptomes from *Ikzf3^+/+^*, *Ikzf3^+/N159S^* and *Ikzf3^N159S/N159S^* mice (n = 2 or 4 per genotype). **(B)** GO analysis of downregulated genes in *Ikzf3^+/N159S^* and *Ikzf3^N159S/N159S^* T and B cells, compared to *Ikzf3^+/+^* controls, with FDR <0.05. Adjusted p values are presented for each GO term. Colors indicate ratio of number of downregulated genes within total number of genes included for each GO term. **(C, D)** Z-score heat maps of downregulated genes in *Ikzf3^N159S/N159S^* B cells **(C)** and T cells (**D**), with the GO term of leukocyte migration included. **(E)** Read counts of Foxo1 in RNA-seq are shown. **(F)** Putative IKZF1 target genes, abstracted by Harmonizome database among the downregulated genes in *Ikzf3^N159S/N159S^* B and T cells. **(G)** UCSC Genome Browser track view of IKAROS ChIP-seq performed in GM12878 cell line (B-lymphocyte) in the ENCODE project, showing the binding by IKAROS around the *SELL* gene. Pool IDR pk is IKZF1 ChIP-seq of GM12878 irreproducible discovery rate (IDR) thresholded peaks pool. Pool foldchg is IKZF1 ChIP-seq of GM12878 fold change over control pool.

To examine what genes were dysregulated in the presence of Aiolos^N159S^ variant, we conducted RNA-seq analyses. Considering low CD62L expression in T cells, we chose CD127 as a marker to isolate cells that would correspond to naïve T cells and purified splenic B220^+^CD19^+^ B cells and TCRβ^+^CD4^+^CD44^−^CD127^+^ T cells of *Ikzf3^+/+^*, *Ikzf3^+/N159S^*, and *Ikzf3^N159S/N159S^* mice. The results obtained from RNA-sequencing (RNA-seq) were analyzed using principal component analysis (PCA). In both B and T cells, PCA showed that *Ikzf3^+/+^*, *Ikzf3^+/N159S^*, and *Ikzf3^N159S/N159S^* cells were differentially clustered in terms of global gene expression (**Figure 6A**). Gene ontology (GO) analysis was subsequently performed using downregulated genes in *Ikzf3^N159S/N159S^* B and T cells. GO analysis indicated that the downregulated genes were enriched in lymphocyte activation, differentiation, and proliferation, leukocyte migration, and cell adhesion (**Figure 6B**). In the heat map, we defined the downregulated genes of leukocyte migration in *Ikzf3^N159S/N159S^* B cells, showing an intermediate phenotype in *Ikzf3^+/N159S^* B cells between WT and homo B cells in terms of gene expression (**Figure 6C**). In *Ikzf3^+/N159S^* T cells, most of genes in the leukocyte migration term also showed an intermediate phenotype, except for *CD24a* and *Cnr2* genes (**Figure 6D**). This observation provides evidence of the gene dosage effect of the Aiolos^N159S^ variant on transcriptional dysregulation. We also examined our RNA-seq data for expression of genes encoding chemokine receptors and found that some chemokine receptors *Ccr6*, *Ccr7*, *Ackr2* and *Ccr1* were downregulated in *Ikzf3^N159S/N159S^* B cells, while only *Cxcr1* showed a minimal increased expression of *Ikzf3^N159S/N159S^* T cells (**Figure S6**).

While CD62L expression in CD4^+^ T cells was not reduced by Aiolos deficiency (50), loss of Ikaros function led to reduced CD62L expression in naïve T cells due to low Foxo1 expression (51). However, Foxo1 expression was not reduced in B or T cells by the *Ikzf3^N159S^* mutation (**Figure 6E**). We have shown that another murine Aiolos variant, Aiolos^G158R^, which has a G to R replacement next to the N159 residue, interferes with Ikaros function (16). To explore the possibility that the genes downregulated by Aiolos^N159S^ were correlated with Ikaros function, we utilized the Harmonizome databases, which is a collection of processed datasets gathered to serve and mine knowledge about genes and proteins from over 70 major online resources (31). Although the Harmonizome database does not have sufficient datasets to predict IKZF3/AIOLOS target genes, IKZF1/IKAROS target genes can be predicted using chromatin immunoprecipitation sequencing (ChIP-seq) datasets of IKZF1 from the ENCODE Transcription Factor Targets datasets. Our analyses of 16 and 31 dysregulated genes in T and B cells, respectively, found that four genes in T cells and more than half of the genes in B cells were associated with IKAROS protein binding (**Figure 6F**). The genome browser track of the ENCODE IKAROS ChIP-seq datasets in the human B cell line showed potential binding of IKAROS to the *SELL* locus encoding CD62L (**Figure 6G**). Thus, transcriptome analyses suggested that the downregulation of some genes by the Aiolos^N159S^ variant was caused by impaired Ikaros function.

### Overexpression of Ikaros restored CD62L expression on Aiolos^N159S/N159S^ B cells

**Figure 7:**
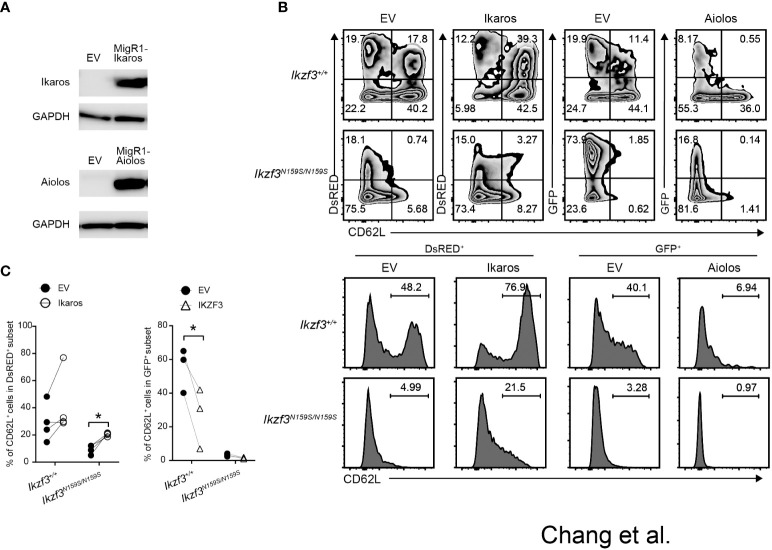
Overexpression of Ikaros restored CD62L expression in *Ikzf3^N159S/N159S^* B cells. **(A)** Immunoblot showing expression of Ikaros and Aiolos protein in packaging Plate E cell line transfected with empty vector (EV), pMigR1-Ikaros or pMigR1-Aiolos vector. **(B)** Zebra plots showing CD62L expression of retrovirally transduced *Ikzf3^+/+^* and *Ikzf3^N159S/N159S^* B cells. DsRED and GFP fluorescent marker expression indicate Ikaros and Aiolos expression, respectively. Lower histograms showing CD62L expression on DsRED^+^ or GFP^+^ transduced cells. Numerical values in each histogram indicate percentages of each region. **(C)** Graphs show the percentage of CD62L^+^ in DsRED^+^ or GFP^+^ subset. Each circle represents data from one experiment. Data are presented as the mean ± SD, n = 3 per group. Student’ s unpaired, two-tailed t test). * P < 0.05.

Given the gene dosage-dependent effect of *Ikzf3^N159S^* on CD62L expression, when low CD62L expression was caused by the interference of Ikaros by Aiolos^N159S^, an increase in the ratio of Ikaros to Aiolos^N159S^ may overcome the dominant effect of the Aiolos^N159S^ variant. To test this possibility, we constructed a retroviral vector encoding either Ikaros with a DsRed marker or Aiolos with a GFP marker. After confirming the presence of Ikaros and Aiolos proteins in the packaging cell line Plat-E by western blotting (WB) (**Figure 7A**), we transduced splenic *Ikzf3^+/+^* and *Ikzf3^N159S/N159S^* B cells by retroviral transduction. Induction with Ikaros significantly increased the frequency of the CD62L^+^ population in *Ikzf3^+/+^* cells. However, Aiolos transduction decreased CD62L expression compared to the empty vector (EV) (**Figures 7B, C**). Importantly, CD62L expression in Ikaros transduced (DsRED^+^) *Ikzf3^N159S/N159S^* B cells was partially restored to approximately 20% (**Figures 7B, C**), although the CD62L expression remained lost after Aiolos transduction. These findings support the hypothesis that Aiolos^N159S^ interferes with Ikaros function, at least in the transcriptional regulating of CD62L expression.

## Discussion

We have previously reported that the patient’s family carrying the heterozygous IKZF3 (c.479 A>G, p.N160S) variant showed impaired T and B cell development (27). A mouse model carrying *Ikzf3^N159S^* mutant allele corresponding to N160S in human AIOLOS showed a similar abnormal immune phenotype in B and T cells, confirming that the I*KZF3^N160S^* is the causal variant of the immune-deficient phenotype in these patients (27). In this study, we investigated the homing capacity of *Ikzf3^N159S/N159S^* lymphocytes and the mechanisms underlying low CD62L expression. Although our previous analyses did not reveal any dominant negative effects on IKAROS by AIOLOS^N160S^ in terms of *in vitro* DNA binding and pericentromeric targeting in the cell line in previous analyses (27), this study revealed that some phenotypes, such as impaired CD62L expression observed in the *Ikzf3^N159S^* mouse model, were probably caused by hijacking of Ikaros function by the Aiolos^N159S^ variant.

Currently, the regulation of the *Sell* gene encoding CD62L remains elusive. Thereby, little is known about how the IKZF family proteins are involved in *Sell* gene regulation. Indu et al. reported that the dominant negative form (IK7) of the Ikaros protein, which lacks the N-terminal DNA-binding ZFs but retains the C-terminal dimerization zinc fingers, disturbs the CD62L expression (52). This suggests that Ik7 Ikaros possibly interferes with *wild-type* (wt) Ikaros protein function necessary for activating *Sell* gene (52). It is conceivable that Ik7 Ikaros influences the binding of Ikzf1 to the regulatory sequences of the *Sell* gene. In this study, we found that two regions in the *SELL* locus are bound by IKAROS in a human B cell line, suggesting that IKAROS directly activates the *SELL* gene. Future studies are required to elucidate the function of these IKAROS-bound regions. In contrast to Ikaros, there have been no reports demonstrating the involvement of Aiolos in CD62L expression. Aiolos-deficient mice showed normal CD62L expression in CD4^+^ T cells (50). Therefore, the Aiolos^N159S^ variant downregulates CD62L expression by interfering with protein functions other than those of Aiolos. Yamashita et al. reported that the Aiolos^G159R^ variant interferes with Ikaros function, causing developmental inhibition of B cells at an early stage in a gene dose-dependent manner (16). Although Aiolos^G159R^ can alter the nuclear localization pattern of Ikaros and *in vitro* binding to the canonical Ikaros site (16), AIOLOS^N159S^ variant did not show such a capacity (27). However, the analyses in this study revealed that some phenotypes observed in *Ikzf3^N159S/N159S^* mice could be caused by interference with Ikaros function. For instance, our RNA-Seq of splenic B and T cells revealed that genes downregulated genes by Aiolos^N159S^ variant included putative targets of Ikaros, such as *Sell*, at least according to the Harmonizome databases. In addition, excessive exogenous Ikaros expression in Aiolos^N159S/N159S^ B cells restored CD62L expression. These findings indicate that the low CD62L expression caused by Aiolos^N159S^ is affected by disruption of Ikaros function. Further elucidation of how Ikaros regulates *Sell* should be conducted in future studies. To date, no IKZF protein variant, other than *Ikzf3^G158R^* and *Ikzf3^N159S^*, has been shown to interfere with other IKZF family members. Interestingly, *IKZF1^N159S^* and recently reported immunodeficiency and multiple system anomalies-causing *IKZF2^G153R^* variants were shown to act dominant-negative against *IKZF1* and *IKZF2*, respectively (15, 53). Thus, it is possible that these pathogenic variants also act against other IKZF proteins, and interference of other IKZF proteins may be partially responsible for the pathogenesis of immunodeficiency caused by these variants.

Parul et al. proposed that the decreased expression of *Sell* due to Ikaros deficiency is mediated by the reduced expression of Foxo1 (51). Although Ikaros transduction of Ikaros-null CD4^+^ T cells upregulated Foxo1 level, CD62L expression remained low. However, Foxo1 transduction increased the expression of CD62L. Parul et al. discussed that increased Ikaros expression might interfere with Foxo1’s ability to activate the *Sell* locus (51). In contrast, in *Ikzf3^N159S/N159S^* B and T cells, Foxo1 level were not reduced, and the transduction of Ikaros into *Ikzf3^N159S/N159S^* B cells partially restored CD62L expression. This discrepancy between Ikaros-null and *Ikzf3^N159S/N159S^* cells suggests that the mechanisms underlying CD62L reduction are different. Further studies are required to address this issue. In contrast to Ikaros, overexpression of *wild-type* Aiolos over endogenous Ikaros inhibited CD62L expression. It is conceivable that excess Aiolos occupies the Ikaros-bound regions in the *Sell* locus. In this case, *Sell* gene was not activated by Aiolos, suggesting that there must be a functional difference between Ikaros and Aiolos in term of the activating of the *Sell* gene after binding to DNA. Both Ikaros and Aiolos are expressed in primary murine lymphocytes; however, the expression level of Aiolos^N159S^ was slightly higher than that of *wt* Aiolos (27). Therefore, it is possible that high amounts of Aiolos^N159S^ cause low CD62L expression, rather than functional differences between Aiolos and Aiolos^N159S^. Interestingly, one among four patients harboring the same N-to-S substitution in the IKZF1/IKAROS protein (*IKZF1^N159S^* variant) showed low CD62L expression (15). Therefore, we speculate that the N to S substitution alters Aiolos function and make Aiolos^N159S^ variant to function as a dominant negative, at least in *Sell* gene regulation.

In mice harboring the *Ikzf3^G158R^* mutation, early B cell development in the bone marrow is inhibited at the pre-Pro B to Pre-B transition, at least in part, by interference with Ikaros function (16). In contrast, early B cell development was mostly normal in *Ikzf3^+/N159S^* and *Ikzf3^N159S/N159S^* mice, with an increased and decreased frequency of recirculating mature B cells in *Ikzf3^+/N159S^* and *Ikzf3^N159S/N159S^* mice, respectively. As Aiolos-deficient mice show a decrease in mature recirculating B cells in the bone marrow (25), the reduction in recirculating B cells in *Ikzf3^N159S/N159S^* mice likely stems from the loss of Aiolos function due to N159S replacement. Similar to recirculating B cells, the frequency of the peritoneal B2 cells was significantly increased in heterozygous mice, while it was decreased in homozygous mice. Thus, as far as these two types of B cells are concerned, *Ikzf3^+/N159S^* and *Ikzf3^N159S/N159S^* mice showed divergent phenotypes. Currently, the mechanisms causing this divergent and cell-specific phenotypes caused by *Ikzf3^N159S^* variant are not yet characterized and need further studies. In addition to the reduction in the splenic B220^+^ population, which consisted mainly of B2 cells, this study revealed that CD5^+^ B1a cells in the peritoneal cavity were nearly lost in *Ikzf3^N159S/N159S^* mice. Given the decrease in the B2/B1 ratio, the loss of CD5^+^ B1a cells likely stems from the developmental inhibition of B1a cells, although our results do not formally exclude the possibility that this is caused by the loss of CD5 expression from B1a cells. A previous study has shown that peritoneal B1a cells are severely reduced in Aiolos-deficient mice (25). In contrast, Ikaros-deficiency increases the number of B1 cells, suggesting a negative role in B1 cell development and function (54, 55). Thus, Ikaros and Aiolos function differently in regulating B1 cell development, suggesting that the loss of CD5^+^ B1a cells in *Ikzf3^N159S/N159S^* mice is likely caused by the loss of Aiolos function. In contrast, CD23 expression in follicular B cells was reduced in Ikaros-deficient mice (54, 56), whereas follicular B cells retained CD23 expression in Aiolos-deficient mice (25). Thus, the loss of CD23 expression in *Ikzf3^N159S/N159S^* splenic B cells was likely caused by the disruption of Ikaros function by Aiolos^N159S^ variant.

The migration of lymphocytes into tissues, a process referred to as lymphocyte homing, is regulated by several molecules. L-selectin (CD62L), which is encoded by *Sell* gene, is an adhesion molecule that binds to ligands expressed on high endothelial venules (HEVs) and plays an important role in maximizing lymphocyte trafficking across HEV (57–60). CD62L^−/−^ lymphocytes failed to enter peripheral lymph nodes across HEV, resulting in a striking reduction in the lymphocyte cellularity in these tissues with a corresponding increase in their cellularity in spleen (57, 60, 61). L-selectin (CD62L) plays a key role in leukocyte migration into lymphoid tissues *via* the regulation of leukocyte rolling and entry into tissues (62). CD62L expression in *Ikzf3^N159S/N159S^* T and B cells was low, and BM chimera experiments showed that those *Ikzf3^N159S/N159S^* cells were outcompeted by control cells, particularly in LNs. These observations suggested that the homing abilities of *Ikzf3^N159S/N159S^* B and T cells were reduced. Indeed, the homing assay confirmed that the ability to enter LNs was weaker in *Ikzf3^N159S/N159S^* B cells than control cells. Unexpectedly, *Ikzf3^N159S/N159S^* cells were nearly undetectable in the bloodstream after injection. In *Ikzf3^N159S/N159S^* mice, the reduction in B cell frequency declined more in the peripheral blood than in the spleen (27). Thus, the reduction of circulating lymphocytes by Aiolos^N159S^ is mediated in part by intrinsic defect in staying in the bloodstream, although the underlying molecular mechanisms for this defect in *Ikzf3^N159S/N159S^* cells are unclear. RNA-seq of B cells detected a decrease in mRNAs-encoding molecules related to cell migration, such as Ccr1, Ccr7 and Slamf1, in *Ikzf3^N159S/N159S^* cells. It is possible that the reduction in these molecules is involved in the decreased frequency of *Ikzf3^N159S/N159S^* cells in the bloodstream. However, these factors are not primarily involved in the low homing capacity of *Ikzf3^N159S/N159S^* cells. Compared to the input *Ikzf3^N159S/N159S^* cells, the frequency of CD62L^+^ cells as well as CD62L expression level were higher in cells that migrated to LNs. This selective enrichment of CD62L^+^ cells in LNs not only confirms the importance of CD62L for lymphocyte homing but also indicates that *Ikzf3^N159S/N159S^* cells retain their homing capacity to LNs once sufficient levels of CD62L are expressed. Therefore, the reduced homing capacity of *Ikzf3^N159S/N159S^* cells is likely attributable to low CD62L expression, although our results did not formally exclude the involvement of other down-regulated molecules for impaired migration capacity of *Ikzf3^N159S/N159S^* cells.

In summary, using a mouse model, we found that *Ikzf3^N159S^* inhibited B1a cell differentiation, and most of immune phenotype occurred in a cell-intrinsic manner. Importantly, reduced CD62L expression by the Aiolos^N159S^ variant is likely caused by hijacking of Ikaros function. The Aiolos^G158R^ variant also interferes with Ikaros function (16), however, the phenotypes caused by *Ikzf3^G158R^* and *Ikzf3^N159S^* mutations are quite different. In terms of CD23 and CD62L expression, *Ikzf3^+/N159S^* cells showed a greater reduction than *Ikzf3^+/G158R^* cells (16, 27). The in silico simulation predicted that G159R replacement pushed the second zinc finger away from DNA (16). Comparison of G159R and N160S by the same in silico simulation predicted that that the N160S mutant experiences reduced stability, higher flexibility, and reduced compactness as a result of loss of key hydrogen bond interactions between the cognate DNA (**Figure S7**). These subtle differences in interactions in the structures might serve as the causative factor for the observed phenotypic effect of the Aiolos^N159S^ mutant as compared to the Aiolos^G158R^ mutant. Thus, amino acid replacement in the residue next to each other differentially affects Aiolos function, and even though these two variants potentially interfere with Ikaros function, the different phenotypes between the two mutant mouse lines suggest that the affected Ikaros target genes should be different. It would be interesting to examine whether the phenotypes observed with *Ikzf3^G158R^* or *Ikzf3^N159S^* were retained, restored, or synergized when the two mutations were combined on one allele by generating an *Ikzf3^G158R:N159S^* mutation. ChIP-seq or interactome studies of *Ikzf3^G158R^* or *Ikzf3^N159S^* variant would be other potential approaches to study differences in molecular pathogenesis between two variants. Currently, little is known about how homo- or heterodimer formation among Ikzf family proteins is controlled, and what target genes are regulated by homo- or heterodimers. To understand the mechanisms that regulate the diversity of Ikzf protein family functions, it is important to explore how homo- or heterodimer formation is regulated.

## Data availability statement

The datasets presented in this study can be found in online repositories. The names of the repository/repositories and accession number(s) can be found below: GSE234649 (GEO).

## Ethics statement

The animal study was approved by Institutional Animal Care and Use Committee of RIKEN Yokohama Branch. The study was conducted in accordance with the local legislation and institutional requirements.

## Author contributions

JC performed experiments, analyzed data, prepared figures, and wrote the manuscript. MY analyzed the RNA-seq data. AP and KZ performed structural analyses. IT supervised the research and wrote the manuscript. All authors contributed to the article and approved the submitted version.
