# Supplementary material for: Impaired tissue homing by the Ikzf3N159S variant is mediated by interfering with Ikaros function
Source: Front Immunol. 2023 Aug 17;14:1239779. doi: 10.3389/fimmu.2023.1239779 (PMC10469740; doi:10.3389/fimmu.2023.1239779)
Supplement: Supplementary file 1 [file DataSheet_1.pdf]

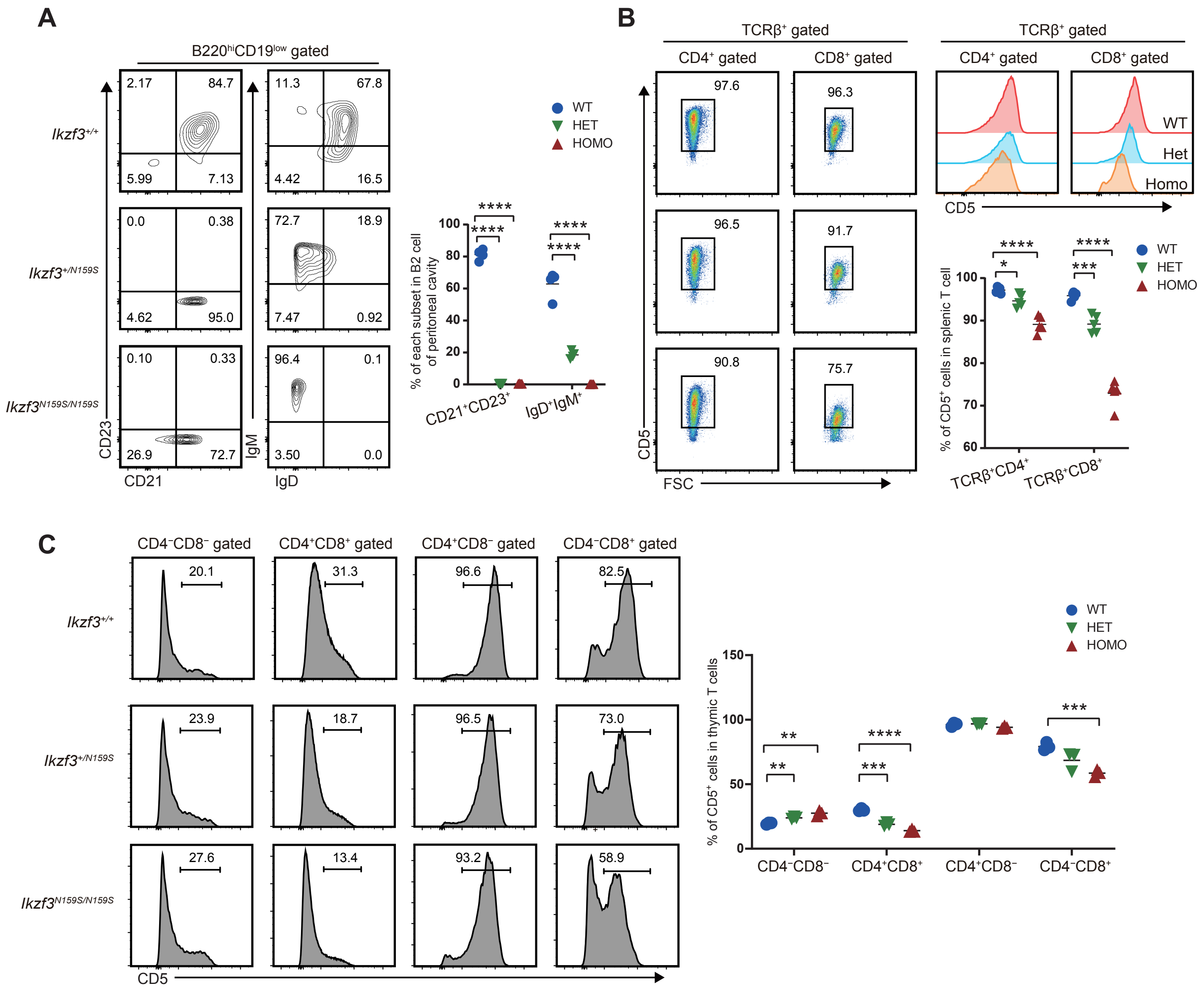

Figure S1. Lymphocyte phenotypes of peritoneal cavity in *Ikzf3*^{N159S} mice. (**A**) FACS analysis of B2 (B220^{hi}CD19^{low}) cell population from peritoneal cavity stained for IgM, IgD, CD21 and CD23 in *Ikzf3*$^{+/+}$, *Ikzf3*$^{+/N159S}$ and *Ikzf3*$^{N159S/N159S}$ mice. Frequencies of IgM^{+}IgD^{+} and CD21^{+}CD23^{+} subsets of B2 cell in peritoneal cavity are shown (n=3). Each dot represents an individual mouse. The horizontal lines indicate the mean values of at least three different mice per group. (**B**) CD5 expressions of splenic CD4^{+} and CD8^{+} T cells are analyzed by flow cytometry. And graph shows the frequency of CD5^{+} cells in splenic CD4 and CD8 T cells. (**C**) CD5 expression of thymic T cells (DN CD4^{-}CD8^{-}, DP CD4^{+}CD8^{+}, CD4 SP CD4^{+}CD8^{-}, CD8 SP CD4^{-}CD8^{+}) is analyzed by flow cytometry. And graph shows the frequency of CD5^{+} cells in thymic T cells. Statistically significant differences (Student' s unpaired, two-tailed t test) between the groups are shown. *, $P < 0.05$; **, $P < 0.01$; ***, $P < 0.001$; ****, $P < 0.0001$.

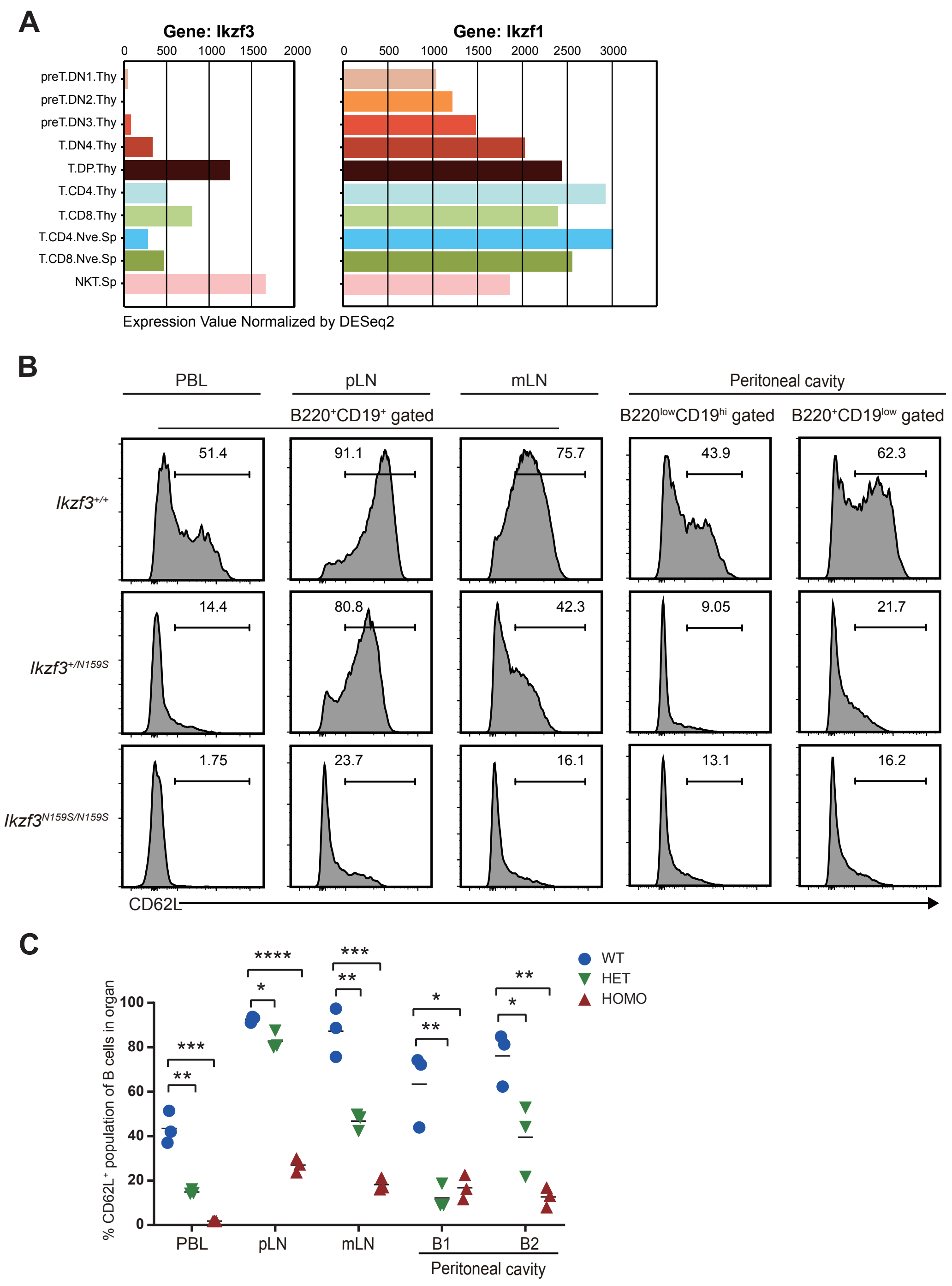

Figure S2. CD62L expression of B cell population in *Ikzf3*^{N159S} mice. (**A**) Expressions Value Normalized by DESeq2 of human thymic and splenic cells are obtained from ImmGen. *Ikzf3* and *Ikzf1* gene expressions are shown in each subsets (preT.DN1.Thy, Lin^{-}CD44^{hi}CD117^{hi}CD25$^{-/lo}$; preT.DN2.Thy, Lin^{-}CD44^{hi}CD117^{hi}CD25^{hi}; preT.DN3.Thy, Lin^{-}CD44^{-}CD25^{hi}; T.DN4.Thy, Lin^{-}CD44^{-}CD25^{-}CD28^{-}; T.DP.Thy, CD4^{+}CD8^{+}TCRβ^{lo}CD69^{-}Dump^{-}; T.CD4.Thy, CD4^{+}CD8^{-}TCRβ^{hi}CD24$^{int/-}$Dump^{-}; T.CD4.Nve.Sp, CD4^{+}CD8^{-}TCRβ^{hi}CD62L^{hi}CD44^{lo}CD25^{-}Dump^{-}; T.CD8.Thy, CD4^{-}CD8^{+}TCRβ^{hi}CD24$^{int/-}$Dump^{-}; T.CD8.Nve.Sp, CD4^{-}CD8^{+}TCRβ^{hi}CD62L^{hi}CD44^{lo}CD25^{-}Dump^{-}; NKT.SP, TCRβ^{Med} CD1d^{+}). (**B**) FACS analyze the CD62L expression of B cell in peripheral blood (PBL), peripheral lymph node (pLN), mesenteric lymph node (mLN) and B1 (CD19^{hi}B220^{lo}), B2 (CD19^{lo}B220^{hi}) cell in peritoneal cavity from *Ikzf3*$^{+/+}$, *Ikzf3*$^{N159S/+}$, *Ikzf3*$^{N159S/N159S}$ mice. (**C**) Graphs show frequencies of CD62L^{+} cell from B220^{+}CD19^{+} B cell (PBL, pLN and mLN), CD19^{lo}B220^{hi} B2 cell (peritoneal cavity) and CD19^{hi}B220^{lo} B1 cell (peritoneal cavity) in *Ikzf3*$^{+/+}$, *Ikzf3*$^{N159S/+}$, *Ikzf3*$^{N159S/N159S}$ (n=3). Each dot represents an individual mouse. The horizontal lines indicate the mean values of at least three different mice per group. Statistically significant differences (Student' s unpaired, two-tailed t test) between the groups are shown. *, $P < 0.05$; **, $P < 0.01$; ***, $P < 0.001$; ****, $P < 0.0001$.

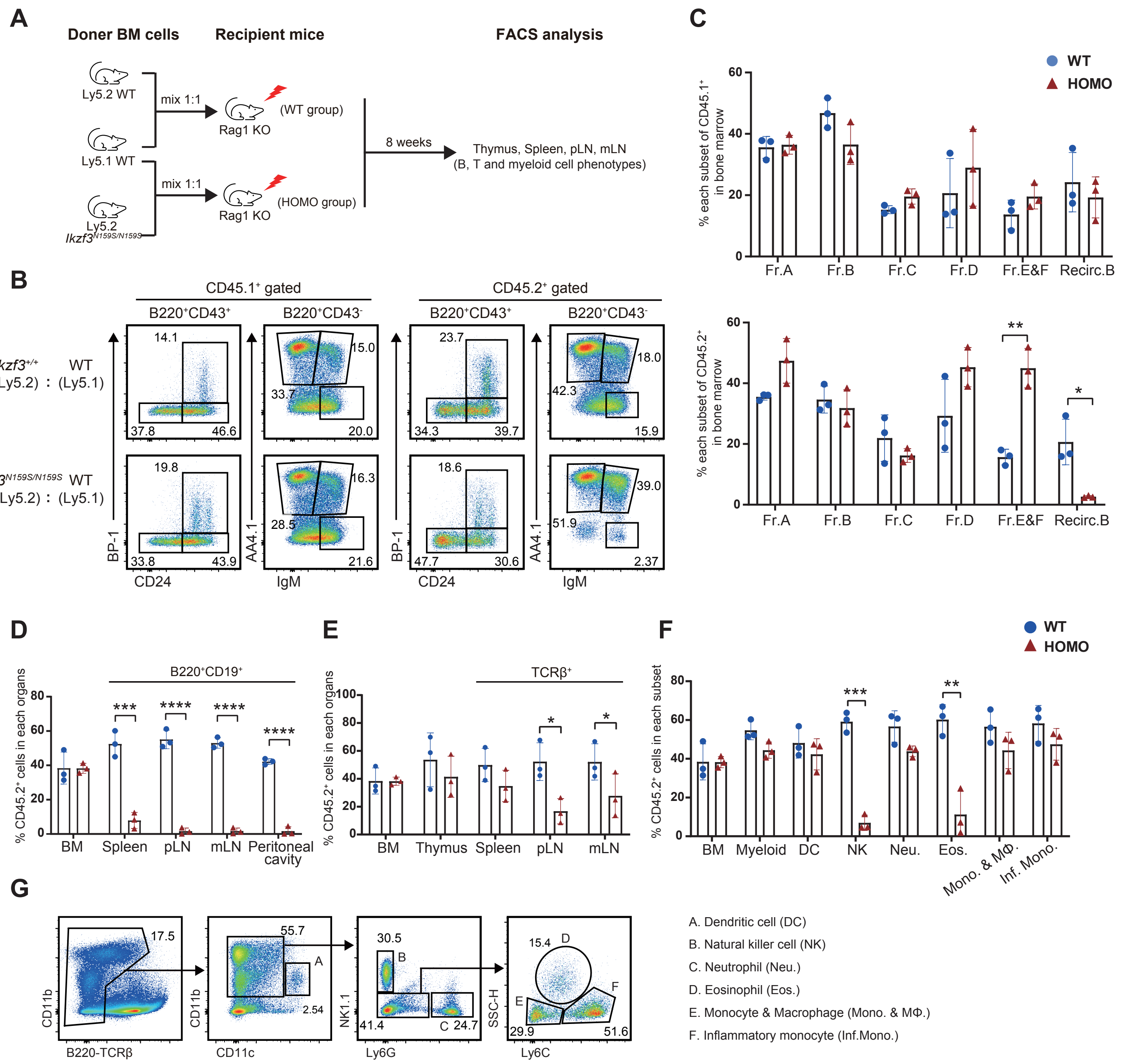

Figure S3. BM chimera experiment using *Ikzf3*$^{+/+}$ and *Ikzf3*$^{N159S/N159S}$ cells. (**A**) CD45.2^{+} *Ikzf3*$^{+/+}$ or *Ikzf3*$^{N159S/N159S}$ BM cells were mixed with CD45.1^{+} wild type BM at a 1:1 ratio and injected to sublethally irradiated Rag1 KO mice. More than 8 weeks after transplantation, flow cytometry analyses were performed. (**B**) Flow cytometric analysis of B cell progenitors' subsets in bone marrow of CD45.1^{+} or CD45.2^{+} subset. (**C**) The frequencies of each B cell rogenitors' subset of D45.1^{+} and CD45.2^{+} bone marrow. Each dot represents an individual mouse. Data are presented as the mean ± SD, n = 3 per group. (**D**) Frequenies of CD45.2^{+} cells of total bone marrow and B220^{+}CD19^{+} cells in each organ from recipients. (**E**) The frequencies of CD45.2^{+} cells in total bone marrow, total thymus, and T cell subset (TCRβ^{+}) of spleen, pLN and mLN. (**F**) The frequencies of CD45.2^{+} cells in total bone marrow and each splenic myeloid subset (myeloid, DC, NK, Neu., Eos., Mono. & MΦ and Inf. Mono.) in spleen. (**G**) Gating strategiesto define myeloid cells subset, dendritic cell (B220^{-}TCRβ^{-}CD11b^{+}CD11b^{-}NK1.1^{-}Ly6G^{+}), eosinophil (B220^{-}TCRβ^{-}CD11b^{+}CD11c^{-}NK1.1^{-}Ly6G^{-}Ly6C^{lo}), Monocyte & Macrophage (B220^{-}TCRβ^{-}CD11b^{+}CD11c^{-}NK1.1^{-}Ly6G^{-}Ly6C^{-}) and Inflammatory monocyte (B220^{-}TCRβ^{-}CD11b^{+}CD11c^{-}NK1.1^{-}Ly6G^{-}Ly6C^{hi}). Student' s unpaired, two-tailed t test), *, $P < 0.05$; **, $P < 0.01$; ***, $P < 0.001$; ****, $P < 0.0001$.

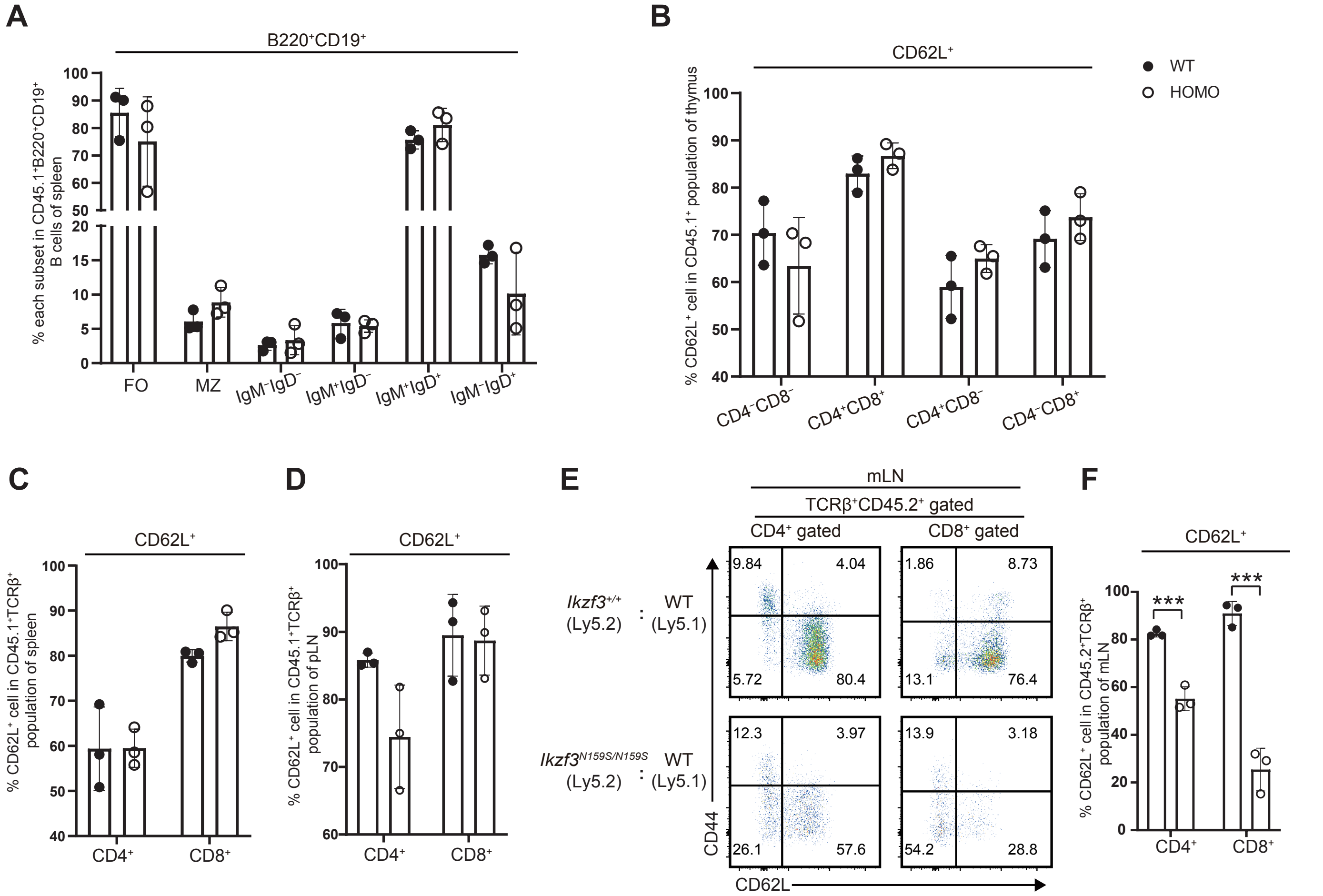

Figure S4. B and T cell phnotypes of CD45.1$^+$ or CD45.2$^+$ *Ikzf3$^{+/+}$* and *Ikzf3$^{N159S/N159S}$* mice in BM chimera experiment. (**A**) The frequencies of IgM$^-$IgD$^-$, IgM$^+$IgD$^-$, IgM$^+$IgD$^+$ and IgM$^-$IgD$^+$ subsets in CD45.1$^+$ B cell population from spleen are shown (n=3). Each dot represents an individual mouse. Data are presented as the mean ± SD, n = 3 per group. (**B**) The frequencies of CD62L$^+$ of CD45.1$^+$ each of thymocyte subset (CD4$^-$CD8$^-$, CD4$^+$CD8$^+$, CD4$^+$CD8$^-$ and CD4$^-$CD8$^+$) (n=3). (**C**) The frequencies of CD62L$^+$ cell of CD45.1$^+$ splenic CD4 and CD8 T cell populations are shown (n=3). (**D**) The frequencies of CD62L$^+$ cell of CD45.1$^+$ CD4 and CD8 T cells in pLN are shown (n=3). (**E**) Flow cytometry showing CD44 and CD62L expression in CD45.1$^+$TCRβ$^+$CD4$^+$, TCRβ$^+$CD8$^+$ cells in mLN. (**F**) The frequencies of CD62L$^+$ cell of CD45.2$^+$ mLN CD4 and CD8 T cell subsets are shown (n=3). Student' s unpaired, two-tailed t test) ***, $P < 0.001$.

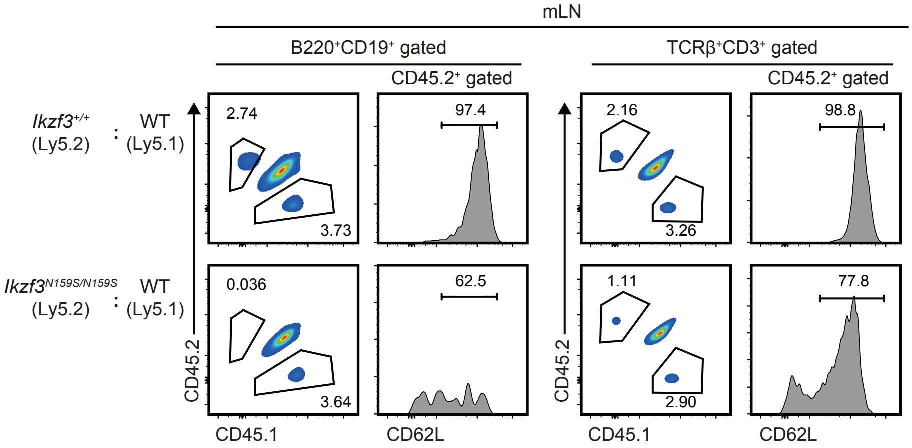

Figure S5. Impaired homing abilities of T cells or B cells of *Ikzf3*$^{N159S/N159S}$ mice. Flow cytometric analysis of the frequency of CD45.2^{+}CD45.1^{-} and CD45.2^{-}CD45.1^{+} in B and T cell population from mLN. Histograms showing CD62L expression of CD45.2^{+} B cell or T cell subset. Representative histograms among three independent experiments.

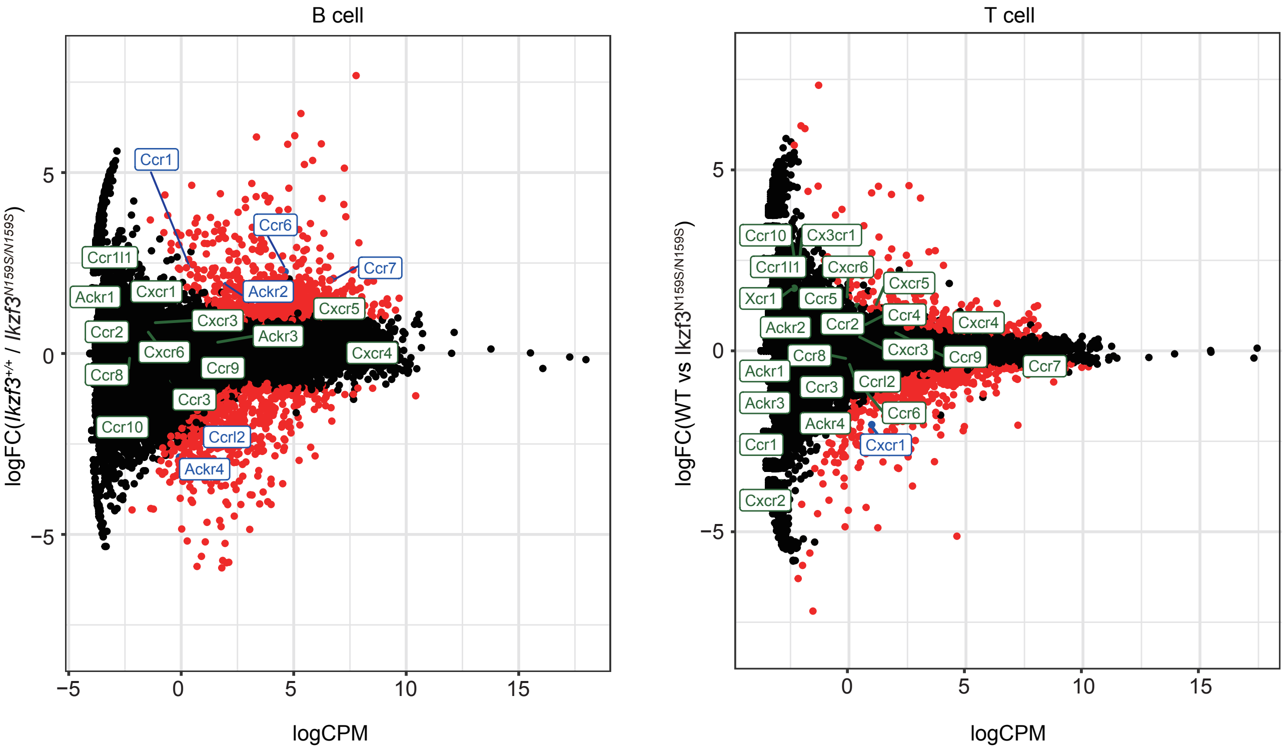

Figure S6. Chemokine receptor expression in B cells and T cells of *Ikzf3*$^{N159S/N159S}$ mice. RNA-seq of splenic B cell and T cell from *Ikzf3*$^{+/+}$ and *Ikzf3*$^{N159S/N159S}$ mice are analyzed. Red dots indicate differentially expressed genes with false discovery rate (FDR) of <0.05. Chemokine receptor genes are annotated. Genes with purple dots and labels are differentially expressed with FDR <0.05, whereas those with green dots and labels are not differentially expressed between the genotyeps. FC, fold change; CPM, counts per million.

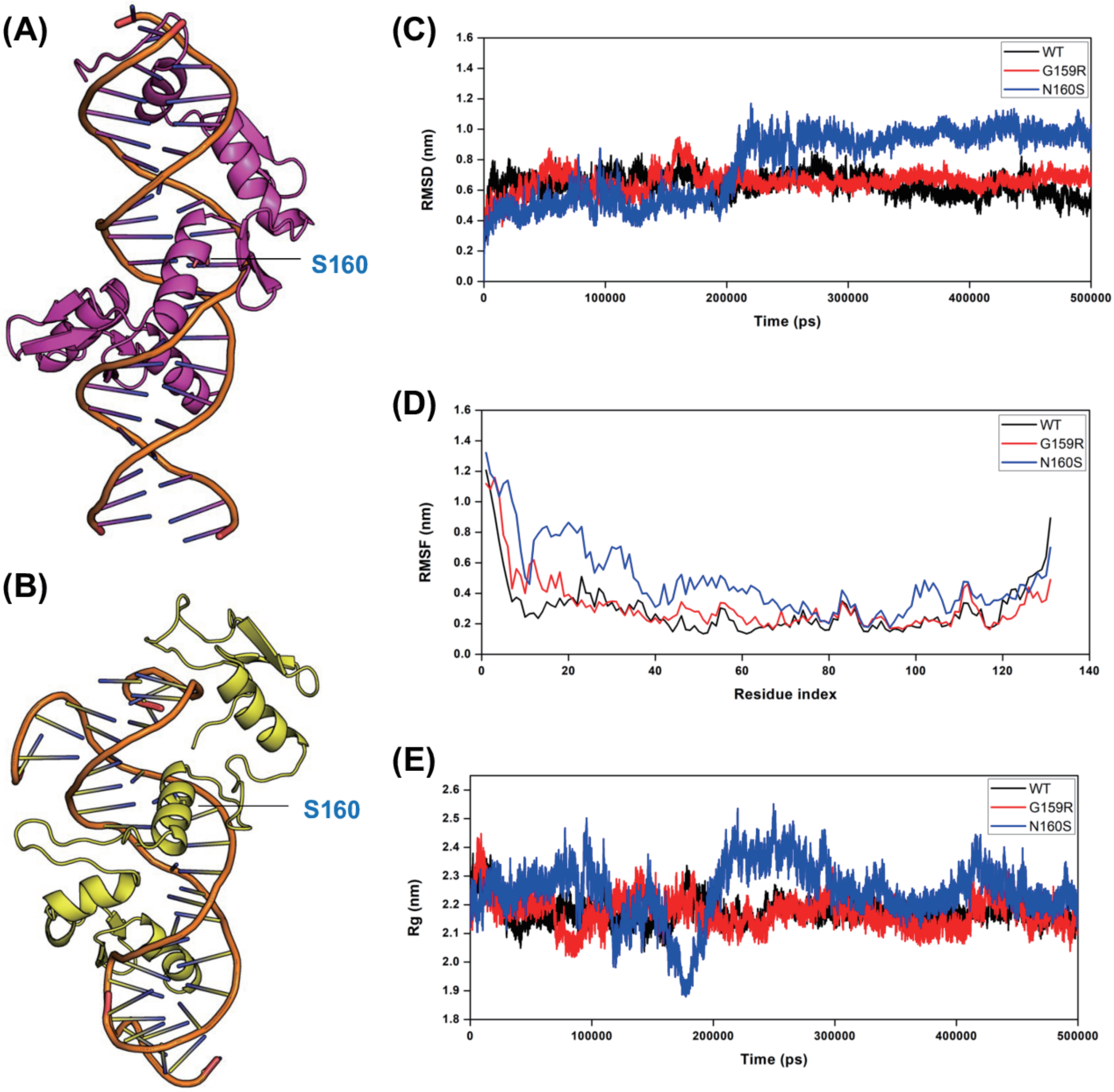

Figure S7. Dynamics and physicochemical properties of WT, G159R and N160S AIOLOS mutants over the entire MD simulation. Analysis with RMSD, RMSF, and Rg. Orientation and conformation of N160S-AIOLOS at the (**A**) beginning and (**B**) during the simulations is shown, where S160 is shown as a stick, (**C**) Plot of time vs. RMSD of the WT, G159R and N160S are shown throughout the MD simulations of 500 ns, (**D**) RMSF plot for WT, G159R and N160S are shown depicting the flexibility of the proteins, and (**E**) Time evolution profiles of the radius of gyration (Rg) for WT, G159R and N160S are shown over the 500 ns simulation time. In all figures, the WT is shown in "black", G159R in "red", and N160S in "light blue".
